# Immunometabolic and epigenetic reprogramming in inflammatory bowel disease: from mechanistic circuits to therapeutic targeting

**DOI:** 10.1186/s40364-026-01037-4

**Published:** 2026-11-06

**Authors:** Yi Han, Jun Yi, Xiaotong Wang, Shizhe Li, Min Chen, Junbo Xiao

**Affiliations:** 1 Department of Gastroenterology, Xiangya Hospital, Central South University, Changsha, Hunan 410008 China; 2 National Clinical Research Center for Geriatric Diseases (Xiangya Hospital of Central South University), Changsha, Hunan 410008 China; 3 Department of Gastroenterology, Zhuzhou Hospital Affiliated to Xiangya School of Medicine, Central South University, Zhuzhou, Hunan China

**Keywords:** Inflammatory bowel disease, Immunometabolism, Epigenetic regulation, Metabolic reprogramming

## Abstract

Inflammatory bowel disease (IBD) is a chronic, immune-mediated disorder of the gastrointestinal tract and an escalating global public health challenge, particularly in newly industrializing regions. Its etiology and pathogenesis remain incompletely understood, although disrupted homeostasis between the intestinal microbiota and the mucosal immune system is implicated. Macrophages and T cells contribute to the balance between tissue injury and repair. Increasing evidence suggests that metabolic state and epigenetic programs interact to influence immune cell function. Under hypoxic and inflammatory stress, glucose, lipid, and amino acid metabolism can undergo remodeling, including shifts between glycolysis and oxidative phosphorylation, altered fatty-acid utilization, changes in tryptophan and methionine metabolism, and fluctuations in methyl-donor availability. Metabolic cues may influence chromatin regulation and immune signaling through acetylation, methylation, lactylation, palmitoylation, phosphorylation, and other modifications. These processes can affect effector programs, the T helper 17–regulatory T cell balance, macrophage inflammatory gene expression, inflammasome activation, and interleukin-10–related signaling, with potential implications for mucosal barrier repair and inflammatory amplification. However, the causal roles of these metabolic and epigenetic alterations in IBD remain to be clarified: they may contribute to disease initiation, arise as accompanying features of the disease state, or amplify inflammation once it is established. This review synthesizes current advances in metabolic reprogramming and epigenetic regulation in macrophages and T cells, examining immunometabolic nodes that link environmental stress to chromatin remodeling. We further summarize emerging therapeutic strategies targeting metabolic and epigenetic pathways, assess evidence from preclinical and clinical studies, and discuss interventions directed at immunometabolic checkpoints.

## Introduction

Inflammatory bowel disease (IBD) is a group of immune-mediated chronic disorders characterized by inflammation of the gastrointestinal tract. Crohn’s disease (CD) and ulcerative colitis (UC) are its two principal phenotypes [1]. In many countries across North America, Oceania, and Europe, the prevalence of IBD now exceeds 0.3 percentage and incidence has plateaued or is declining. In contrast, newly industrialized regions in Africa, Asia, and South America continue to experience rising incidence [2]. These divergent trends underscore the need for preventive research and innovation in health system design to contend with a complex and resource-intensive condition. Although the precise etiology remains unresolved, current evidence supports a central role for disrupted homeostasis between the resident intestinal microbiota and the mucosal immune system [3]. Loss of epithelial tight junction integrity, compromise of the mucus layer, and alteration of antimicrobial peptide profiles together increase exposure to antigens and microbe-associated molecular patterns, amplifying cascades in both innate and adaptive immunity. Data from animal models and human cohorts indicate that barrier dysfunction can directly trigger or markedly exacerbate intestinal inflammation [4–7]. On this basis, international consensus statements advocate coordinated treatment targets that include clinical remission, endoscopic mucosal healing, and improvement of biomarkers, with sustained monitoring and intervention at the level of mucosal immunity to improve long term outcomes [8].

Multiple immune cell populations contribute to IBD pathogenesis, including B cells [9], dendritic cells [10], neutrophils [11], and natural killer cells [12]. This review focuses on macrophages and T cells, whose metabolic states and epigenetic programs shape their functions during mucosal inflammation. Studies in immunometabolism show that remodeling of mitochondrial function, glycolysis, and oxidative metabolism influences the activation and persistence of innate and adaptive immune cells. In IBD, these findings have prompted investigation of interventions targeting metabolic pathways [13]. Metabolic and epigenetic regulation intersect in both populations. At the epigenetic level, the microbial metabolite butyrate inhibits histone deacetylases (HDACs), enhances acetylation at the forkhead box P3 (Foxp3) locus, and promotes colonic regulatory T-cell (Treg) differentiation. It also attenuates inflammatory responses in intestinal macrophages [14, 15]. In T cells, mechanistic target of rapamycin (mTOR) integrates nutrient availability with T-cell receptor (TCR) and cytokine signals to regulate glycolysis, oxidative metabolism, and the differentiation and function of effector and regulatory cells [16]. These findings provide the basis for examining how metabolic and epigenetic changes influence macrophage responses, T-cell function, and the balance between intestinal inflammation and repair.

Unlike prior reviews that have mainly discussed immunometabolism, epigenetic regulation, post-translational modifications, or biomarkers as separate themes, this review integrates these layers into a macrophage-T cell-centered framework. We examine whether metabolic-epigenetic coupling contributes to relapse-prone immune states, identify associated molecular signatures, and discuss its implications for biomarker-guided therapeutic targeting in IBD. In this review, we first outline how the epithelial barrier and innate sensing pathways set antigen exposure and immune thresholds. We then synthesize how metabolic reprogramming and epigenetic regulation in macrophages and T cells shape inflammatory intensity and tissue repair. Finally, we discuss the translational potential of metabolic and epigenetic targets and propose strategies to align these approaches with current therapeutic endpoints.

## Search strategy and selection criteria

We searched PubMed, Web of Science, and Scopus for English-language publications from database inception to September 2026. Searches combined terms for inflammatory bowel disease (“inflammatory bowel disease”, “Crohn’s disease”, and “ulcerative colitis”) with terms related to macrophages, monocytes, T cells, immunometabolism, metabolic reprogramming, and epigenetic regulation. Additional terms included “acetylation”, “methylation”, “lactylation”, “palmitoylation”, “phosphorylation”, and “inflammatory memory”. Relevant terms and their synonyms were combined using AND/OR.

Articles were selected for their relevance to metabolic and epigenetic regulation of macrophage and T-cell function, intestinal inflammation, and therapeutic targeting. We considered original studies in experimental models and human populations, together with relevant reviews, prioritizing research that connected molecular mechanisms with functional outcomes. Studies outside IBD were included when they informed mechanisms discussed in this review, with their experimental context specified. Additional publications were identified from the reference lists of selected articles.

## Architecture of the immune microenvironment in IBD

The balance between mucosal inflammation and immune tolerance in IBD depends on interactions among multiple cell types. This balance is shaped by host genetic susceptibility and long-term interactions with the intestinal microbiota [17, 18]. Within this network, lamina propria monocytes and macrophages sense signals from microbes and damaged tissue and link events at the epithelial–microbial interface to adaptive responses mediated by T and B cells [19, 20]. Under homeostatic conditions, mucosal phagocytes expressing high levels of C-X3-C motif chemokine receptor 1 (CX3CR1) extend dendrites across the epithelium to sample luminal antigens. This process contributes to pathogen clearance and provides a foundation for immune tolerance [21]. In mouse models, inflammation triggers the recruitment of circulating Ly6C^hi^ monocytes into the intestinal wall through a process dependent on CC chemokine receptor 2 (CCR2). These monocytes differentiate into proinflammatory effector cells that reshape the local cytokine and metabolic environment and promote lesion progression [22]. Interleukin-10 (IL-10) signaling through the IL-10 receptor (IL-10R) restrains the proinflammatory responses of these myeloid cells. Studies of patients with monogenic very early-onset IBD demonstrate the importance of this pathway in intestinal immune regulation [23, 24].

### Macrophage ontogeny, functional programming, and crosstalk at the epithelial interface

**Fig. 1:**
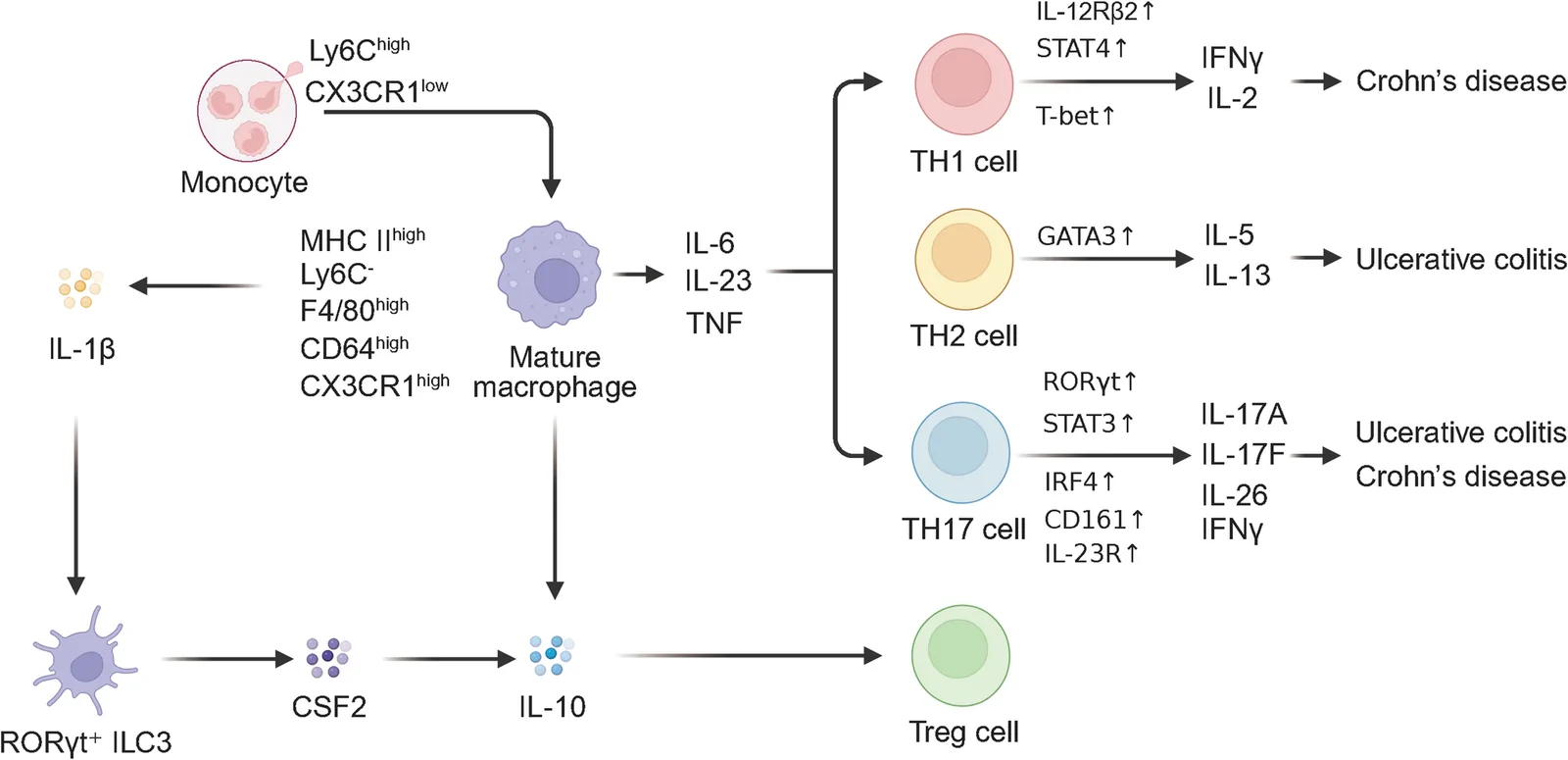
IBD macrophages and T cells in the intestinal immune circuit. In mice, circulating Ly6Chi CX3CR1low monocytes enter the lamina propria and progressively differentiate, acquiring MHC II expression, losing Ly6C, and upregulating F4/80, CD64, and CX3CR1. In mouse studies, macrophage-derived IL-10 supports Treg expansion in experimental oral-tolerance settings. Microbiota-induced macrophage IL-1β also stimulates GM-CSF/CSF2 production by ILC3s. GM-CSF regulates the functions of intestinal myeloid cells and contributes to Treg homeostasis. In Crohn’s disease, T cells produce increased IFN-γ and IL-2 and upregulate IL-12Rβ2, STAT4, and T-bet, indicating active TH1 differentiation. In ulcerative colitis, TH2-associated pathways are more prominent: T cells produce IL-5 and IL-13 and express GATA3. IL-13 promotes fibrosis, disrupts epithelial tight junctions, and induces epithelial apoptosis, thereby driving ulcer formation. The TH17 axis is active in both forms of IBD; T cells show elevated IL-17A and IL-17F and can also produce IL-26 and IFN-γ, and the associated cells express CD161, IL-23R, RORγt, STAT3, and IRF4

Under homeostatic conditions, the colonic macrophage pool in mice is maintained mainly by the continuous, CCR2-dependent recruitment of circulating Ly6C^hi^ CX3CR1^low^ monocytes. After entering the lamina propria, these monocytes progressively differentiate into mature tissue-resident macrophages, first acquiring major histocompatibility complex class II (MHC II) expression, then losing Ly6C, and subsequently upregulating F4/80, CD64, and CX3CR1 (Fig. 1) [25, 26]. IL-10–IL-10R signaling is central to maintaining a regulatory macrophage phenotype. Myeloid-specific deletion of IL-10 does not cause spontaneous colitis in mice [27]. In contrast, macrophage-specific loss of IL-10R promotes a proinflammatory phenotype, with increased expression of inducible nitric oxide synthase (iNOS), interleukin-23 (IL-23), and interleukin-12 (IL-12), and leads to spontaneous colitis [28, 29]. In patients with early-onset severe colitis, loss-of-function variants in IL10RA or IL10RB impair IL-10–dependent signal transducer and activator of transcription 3 (STAT3) activation and compromise the control of proinflammatory responses [23]. These monogenic forms of IBD illustrate the consequences of impaired IL-10 signaling for intestinal immune homeostasis [23, 24]. Together, the human genetic findings and experimental myeloid-cell studies support a role for IL-10R signaling in controlling intestinal inflammatory responses.

At the intracellular level, loss of IL-10 signaling reduces STAT3 activation [30] and disrupts epigenetic mechanisms that normally restrict chromatin accessibility at proinflammatory genes [31, 32]. Loss of this signaling also reduces the expression of IκBNS, a negative regulator of nuclear factor κB (NF-κB) [32]. IL-10 also induces DNA damage-inducible transcript 4 (DDIT4), which restrains lipopolysaccharide (LPS)-induced metabolic reprogramming by limiting glucose uptake and glycolysis while supporting oxidative phosphorylation (OXPHOS) [33]. These IL-10-dependent mechanisms link transcriptional, epigenetic, and metabolic regulation to the control of macrophage inflammatory responses.

Macrophages also contribute to epithelial barrier defense through their localization near the epithelium and interactions with luminal microbes. These cells typically express high levels of CX3CR1 and are found in regions enriched in epithelial-derived C-X3-C motif chemokine ligand 1 (CX3CL1). CX3CL1–CX3CR1 signaling regulates macrophage differentiation and function [34]. Disruption of CX3CL1–CX3CR1 signaling exacerbates dextran sulfate sodium (DSS)-induced colitis [35] and reduces IL-10 production by macrophages, thereby impairing support for Treg expansion in the lamina propria [36]. CX3CR1-positive macrophages extend transepithelial dendrites to sample luminal antigens and bacteria [37, 38]. During early infection, these cells can migrate into the intestinal lumen and encounter invasive pathogens such as *Salmonella enterica* serovar Typhimurium [37, 38]. In patients with CD, a missense variant in CX3CR1 has been associated with impaired antifungal responses [39].

Macrophage interactions with intestinal microbes also shape mucosal lymphocyte responses through IL-1β and IL-10. Dysregulated inflammasome activation increases IL-1β production and amplifies inflammation. Under homeostatic conditions, however, microbiota-induced IL-1β production by macrophages contributes to a regulatory circuit involving group 3 innate lymphoid cells (ILC3s) [33, 40]. Macrophage-derived IL-10 supports the maintenance and expansion of antigen-specific Tregs in the intestinal mucosa of mice [36]. In the macrophage-ILC3 circuit, IL-1β stimulates ILC3 production of granulocyte-macrophage colony-stimulating factor (GM-CSF; also termed CSF2). GM-CSF regulates the functions of intestinal myeloid cells, including macrophages, and supports Treg homeostasis [36, 40].

### T-cell programs that balance injury and repair

T cells accumulate in inflamed intestinal tissue and contribute to local inflammatory cytokine production. Their role in sustaining intestinal inflammation is also supported by T-cell-dependent models of chronic colitis [41]. Lamina propria T cells from patients with IBD respond weakly to TCR stimulation and depend on survival signals from interleukin-6 (IL-6) and tumor necrosis factor (TNF). Their cytokine production is regulated by transcription factors including nuclear factor of activated T cells 2 (NFAT2) and interferon regulatory factor 4 (IRF4) [42, 43]. The following paragraphs examine T helper 1 (Th1), T helper 2 (Th2), and T helper 17 (Th17) responses in intestinal inflammation.

In CD, T cells from the lamina propria and mesenteric lymph nodes produce more interferon gamma (IFN-γ) and interleukin-2 (IL-2) and upregulate interleukin-12 receptor beta 2 (IL-12Rβ2), STAT4, and T-box transcription factor 21 (T-bet; encoded by *TBX21*), consistent with T helper 1 (Th1) differentiation [44]. In mouse models of colitis, STAT4 deletion is protective, whereas its overexpression exacerbates disease [45]. However, in a phase II trial in CD, IFN-γ blockade with fontolizumab did not improve the primary endpoint of clinical response at day 29 compared with placebo [46].

Pathways related to T helper 2 (Th2) differentiation are more prominent in UC [47]. In UC, lamina propria T cells produce interleukin-5 (IL-5) and interleukin-13 (IL-13) and express GATA-binding protein 3 (GATA3). However, interleukin-4 (IL-4) production remains low, consistent with a nonclassical Th2 response [48, 49]. In some patients with UC, CD1d-restricted nonclassical natural killer T cells produce high levels of IL-13 [48]. Functionally, IL-13 promotes fibrosis, disrupts epithelial tight junctions, and induces epithelial apoptosis, thereby driving ulcer formation. In oxazolone-induced colitis, neutralizing IL-13 or blocking IL-25 signaling reduces intestinal inflammation [50, 51]. These observations motivated clinical trials of anti-interleukin 13 agents such as anrukinzumab and tralokinumab. However, increased mucosal IL-13 has not been consistently observed across studies. In a phase II trial, tralokinumab did not meet the primary endpoint of clinical response, indicating that strategies targeting IL-13 alone require further evaluation [52, 53].

Microbiota-derived signals promote intestinal Th17 differentiation in mouse studies [54]. Experimental work has also identified lamina propria dendritic cells in the terminal ileum as a source of IL-23 [55]. Mucosal T cells from patients with IBD show increased production of interleukin-17A (IL-17A) and interleukin-17F (IL-17F) and can also produce interleukin-26 (IL-26) and IFN-γ. Interleukin-21 (IL-21), IL-23, and TNF-like ligand 1A (TL1A; encoded by TNFSF15) support the induction or maintenance of Th17 responses. These Th17-associated cells express CD161, IL-23 receptor (IL-23R), retinoic acid receptor-related orphan receptor γt (RORγt), STAT3, and IRF4 [56]. IL-17 and IL-21 increase the expression of TNF, IL-1β, IL-6, and interleukin-8 (IL-8), promote neutrophil recruitment, and stimulate matrix metalloproteinase secretion by fibroblasts. IL-26 further amplifies inflammatory responses [57, 58]. Interleukin-22 (IL-22) promotes epithelial proliferation and repair through STAT3 and induces the expression of defensins, mucins, and REG3-family proteins [59, 60]. T helper 22 (Th22) cells are a source of IL-22, and reduced Th22 cell abundance has been reported in actively inflamed mucosa from patients with UC [59, 60]. Recombinant IL-22 ameliorates DSS-induced acute colitis in mice [61]. In a clinical trial in CD, IL-17A blockade with secukinumab did not demonstrate clinical efficacy and was associated with more adverse events [56]. These findings illustrate that the effects of individual cytokines cannot be inferred solely from their association with a particular T-cell subset.

Macrophage recruitment, tissue positioning, and functional state influence local T-cell responses, while T-cell-derived signals help shape the intestinal inflammatory environment. These interactions provide the cellular context for the metabolic and epigenetic mechanisms discussed in the following sections.

## Metabolic reprogramming and immune control in IBD

Obesity associates with human intestinal inflammation [62]. In mice, excessive exposure to macronutrients or food additives can trigger or worsen intestinal inflammation and promote innate immune responses locally and systemically [63]. These observations suggest complex interactions between metabolic dysregulation and mucosal immune responses. Further work shows that genetic risk for CD affects purine metabolism in macrophages, a process required for their inflammatory responses during infection [64], a concept framed as immunometabolism [13].

### Glucose pathways that set inflammatory thresholds

**Fig. 2:**
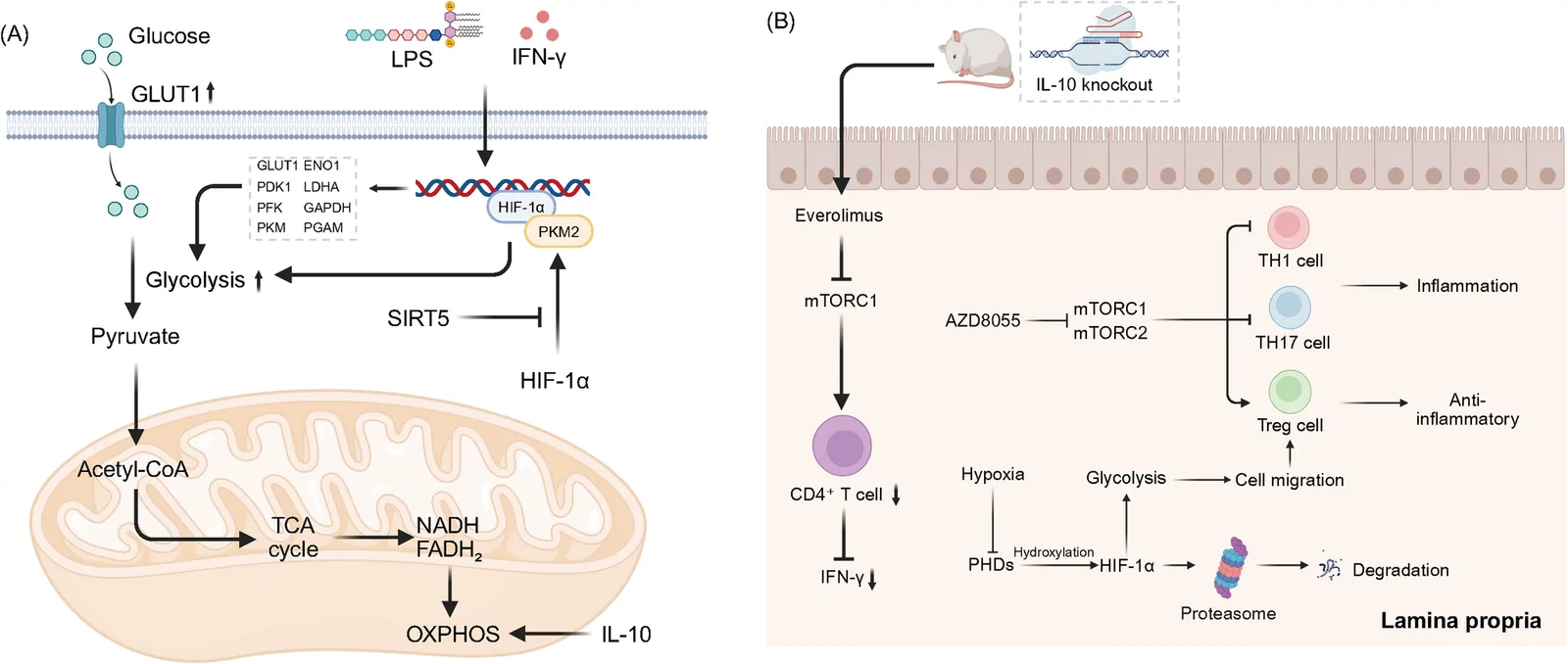
Glucose metabolic reprogramming of macrophages and T cells in IBD. (**A**) At inflamed intestinal sites, inflammatory macrophage states can show increased reliance on aerobic glycolysis relative to oxidative metabolism. In LPS/IFN-γ-stimulated BMDMs, the mRNA levels of genes involved in glucose transport and glycolysis, including GLUT1, ENO1, PKM, PDK1, ALDO, LDHA, PGAM, PFK, and GAPDH, is markedly increased, consistent with enhanced glycolytic activity under these experimental conditions. HIF-1α–mediated glycolytic reprogramming occurs via nuclear PKM2/HIF-1α complex formation. SIRT5-mediated desuccinylation of PKM2 suppresses macrophage IL-1β production, thereby preventing DSS-induced colitis. IL-10 promotes OXPHOS and enhances mitochondrial function in macrophages. (**B**) In an IL-10^−/−^ colitis model, Everolimus blockade of mTORC1 activity is protective, reducing infiltration of CD4^+^ T cells in the colonic LP and lowering IFN-γ production, thereby ameliorating disease. The dual mTORC1/mTORC2 inhibitor AZD8055 promotes Treg differentiation while suppressing Th1 and Th17 responses and the production of proinflammatory cytokines. HIF-1α-dependent effects on Treg metabolism, migration, and suppressive function vary across experimental settings. Oxygen-sensing PHDs regulate HIF-1α stability: under normoxia, PHDs hydroxylate HIF-1α and target it for proteasomal degradation, whereas hypoxia limits PHD activity and allows HIF-1α accumulation. Glycolysis initiates Treg migration via a PI3K–mTORC2 pathway that induces GCK; GCK binds ACTIN to drive cytoskeletal remodeling, thereby promoting the trafficking of activated Tregs to inflamed tissues to exert immunoregulatory functions. IBD, inflammatory bowel disease; OXPHOS, oxidative phosphorylation; BMDM, bone marrow–derived macrophage; GLUT1, glucose transporter 1; ENO1, enolase 1; PKM, pyruvate kinase M; PKM2, pyruvate kinase M2; PDK1, pyruvate dehydrogenase kinase 1; ALDO, aldolase; LDHA, lactate dehydrogenase A; PGAM, phosphoglycerate mutase; PFK, phosphofructokinase; mTORC1/2, mechanistic target of rapamycin complex 1/2; PHDs, prolyl hydroxylases; PI3K, phosphoinositide 3-kinase; GCK, glucokinase; GAPDH, glyceraldehyde-3-phosphate dehydrogenase; SIRT5, sirtuin 5

Immune cell metabolism is pivotal for intestinal homeostasis. Hypoxia-inducible factor 1 alpha (HIF-1α) and mTOR regulate glycolysis and macrophage functions [65]. Inhibiting HIF-1α or mTOR in macrophages ameliorates disease in colitis models [66, 67]. Interleukin 10 suppresses macrophage activation in colitis by blocking mTOR activity and glycolysis [33]. Interleukin 10 also enhances mitochondrial function by promoting oxidative phosphorylation (OXPHOS) and facilitating mitophagy of damaged mitochondria [33]. Once intestinal inflammation is established, an imbalance between local oxygen supply and consumption can produce pronounced hypoxia at inflamed sites [68]. Within this inflammatory environment, macrophages can increase their reliance on glycolysis (Fig. 2A) [69]. Inflammatory hypoxia and associated HIF-1α signaling can then reinforce proinflammatory myeloid responses, forming a feedback loop that amplifies ongoing inflammation [70]. Lactate derived from glycolysis modulates macrophage polarization, and the NLRP3 inflammasome mediates polarization in innate immunity. In macrophage experiments, inhibition of lactic acid fermentation reduced NLRP3 inflammasome activation, whereas inhibition of mitochondrial pyruvate oxidation enhanced it [71]. In mouse bone marrow-derived macrophages (BMDMs) stimulated with LPS and IFN-γ, messenger RNA levels of glycolytic enzymes increase [72]. Tiliroside promotes HIF-1α degradation and suppresses glycolysis in LPS/IFN-γ-stimulated mouse macrophages, reducing inflammatory activation and attenuating chemically induced colitis in mice [73]. HIF-1α-mediated glycolytic reprogramming is regulated by pyruvate kinase M2 (PKM2), which forms a nuclear PKM2 HIF-1α complex [74]. Desuccinylation of PKM2 mediated by sirtuin 5 (SIRT5) suppresses macrophage IL-1β production and protects against DSS-induced colitis [75].

In active IBD, proinflammatory T cells increase in inflamed tissue whereas Treg function is impaired [76]. Targeting T cell metabolism can modulate differentiation, migration, and survival independently of antigen specificity [77]. The mTOR complex 1 (mTORC1) pathway controls glycolysis and thereby T cell function (Fig. 2B) [16]. In interleukin 10 knockout colitis, the mTOR inhibitor everolimus ameliorates established colitis, reducing lamina propria CD4 positive T cell infiltration and decreasing IFN-γ production [78]. Dual inhibition of mTORC1 and mTOR complex 2 (mTORC2) by AZD8055 promotes Treg differentiation while suppressing Th1 and Th17 responses and proinflammatory cytokines, which mitigates colitis in animals [79]. These findings link mTOR-dependent metabolic control to the balance between inflammatory T-cell responses and Treg differentiation. HIF-1α-dependent metabolic effects vary with the T-cell state and disease model. In a murine glioblastoma study, HIF-1α supported glycolytic Treg migration, whereas HIF-1α-deficient Treg showed increased mitochondrial pyruvate utilization and suppressive activity [80]. HIF-1α also has context-dependent effects in intestinal inflammation. In a mouse study, T-cell-specific HIF-1α deficiency increased susceptibility to DSS-induced colitis [81]. Prolyl hydroxylases (PHDs) regulate HIF-1α stability. Under normoxic conditions, PHDs hydroxylate HIF-1α to mark it for proteasomal degradation, whereas hypoxia limits PHD activity and allows HIF-1α accumulation. Targeting PHDs to stabilize HIF-1α may promote Treg differentiation, enhance anti-inflammatory programs, and treat intestinal inflammation [81]. This oxygen-sensing pathway therefore links metabolic adaptation to Treg differentiation and suppressive function. Activated Treg must migrate to inflamed sites to exert control, and this migration depends on glycolysis [82]. Glycolysis initiates migration through a phosphoinositide 3-kinase (PI3K)/mTORC2 pathway that induces glucokinase (GCK). GCK binds actin to drive cytoskeletal remodeling and increase motility [83]. Together, these studies show that glucose metabolism regulates T-cell differentiation, suppressive function, and migration through interconnected mTOR-, HIF-1α-, and GCK-related pathways.

Glucose metabolism therefore influences both macrophage activation and T-cell function. Hypoxia-associated metabolic changes can reinforce inflammation after intestinal injury has developed, but the effects of targeting these pathways depend on the responding cell population and experimental context.

### Lipid circuits that shape macrophage and T-cell fate

**Fig. 3:**
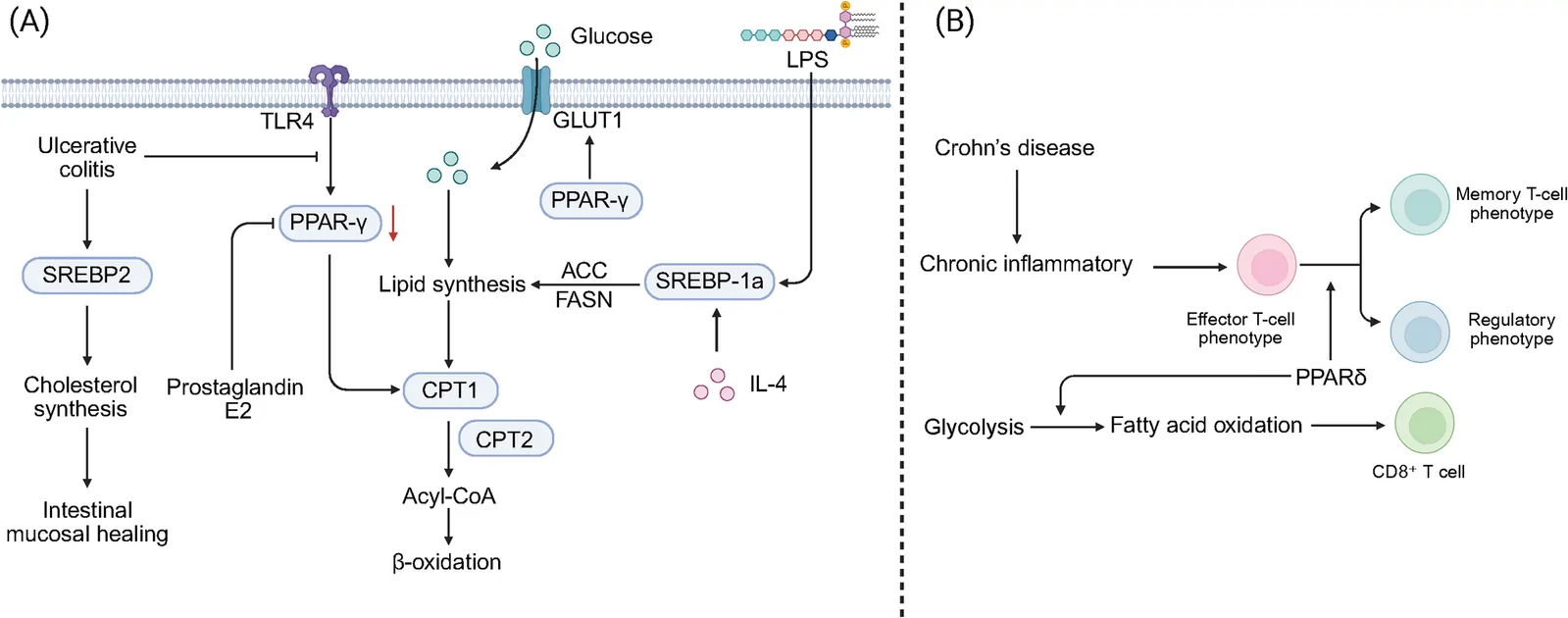
PPAR-driven lipid metabolic reprogramming in macrophages and T cells. (**A**) PPARγ enhances glucose uptake by upregulating GLUT1, supplying substrate for lipogenesis. PPARγ agonists markedly increase CPT1 mRNA. In human THP-1-derived and mouse bone marrow-derived macrophages stimulated with IL-4 and IL-13, AA attenuates the cytokine-induced alternative-activation phenotype, whereas PGE2 enhances this phenotype and mitochondrial OXPHOS in association with PPARγ inhibition. Reduced PPARγ expression in UC was reported in colonic epithelial cells. SREBP drives lipid accumulation; SREBP-1a regulates two key lipogenic enzymes, ACC and FASN. In LPS-stimulated macrophage models, SREBP-1a supports lipid synthesis and inflammatory cellular responses. In IL-4-stimulated mouse macrophages, SREBP1-dependent fatty acid synthesis supports alternative activation. (**B**) Long-term persistence of memory CD8 + T cells is supported by FAO. During memory formation, PPARδ promotes oxidative metabolic programs and memory-cell persistence. The therapeutic relevance of this process to CD remains to be established; memory-cell persistence does not itself demonstrate anti-inflammatory or regulatory function. PPARγ, peroxisome proliferator–activated receptor gamma; GLUT1, glucose transporter 1; CPT1, carnitine palmitoyltransferase 1; AA, arachidonic acid; PGE2, prostaglandin E2; OXPHOS, oxidative phosphorylation; UC, ulcerative colitis; TLR4, Toll-like receptor 4; IBD, inflammatory bowel disease; FAO, fatty acid oxidation; SREBP, sterol regulatory element–binding protein; SREBP-1a, sterol regulatory element–binding protein-1a; ACC, acetyl-CoA carboxylase; FASN, fatty acid synthase; M1, classically activated macrophage; LPS, lipopolysaccharide; SREBP2, sterol regulatory element–binding protein 2; CD, Crohn’s disease; PPARδ, peroxisome proliferator-activated receptor delta

Peroxisome proliferator activated receptor gamma (PPARγ) is a key immunometabolic regulator in IBD [84]. PPARγ regulates macrophage lipid metabolism, including fatty acid oxidation (FAO), and can support anti-inflammatory functions such as interleukin 10 production [85]. In a DSS-induced mouse colitis model, pharmacological PPARγ activation was associated with increased STAT6 phosphorylation and changes in colonic macrophage-associated markers, including lower iNOS and higher arginase 1 (Arg1), Fizz1, and Ym1 expression [86]. Mechanistically, PPARγ upregulates GLUT1 to increase glucose uptake and supply substrates for lipogenesis, thereby coordinating glucose lipid metabolic control (Fig. 3A) [87]. PPARγ agonists such as troglitazone markedly increase carnitine palmitoyltransferase 1 (CPT1) messenger RNA [88]. Myeloid-specific Cpt2 deletion impaired macrophage fatty acid oxidation but did not prevent IL-4-induced expression of the M2-associated markers CD206 and CD301 in BMDMs or peritoneal macrophages [89]. Here, M1/M2 terminology refers to the experimental activation conditions or marker profiles reported in the cited studies, rather than a binary classification of macrophages in human IBD [90–92]. In human and mouse macrophages, inflammatory cytokines including IFN-γ and tumor necrosis factor alpha (TNF-α) reduce PPARγ expression and activity [93]. In human THP-1-derived macrophages and mouse bone marrow-derived macrophages stimulated with IL-4 and IL-13, arachidonic acid attenuated the cytokine-induced alternative-activation phenotype, whereas its metabolite prostaglandin E2 (PGE2) enhanced this phenotype by inhibiting PPARγ and increased mitochondrial OXPHOS [94]. Together, these findings link PPARγ-associated lipid metabolism to macrophage polarization, mitochondrial activity, and inflammatory function.

In UC, reduced PPARγ expression has been reported in colonic epithelial cells in both inflamed and noninflamed mucosa [95]. In DSS-treated mice carrying Toll-like receptor 4 (TLR4) variants, impaired PPARγ signaling and reduced expression of markers associated with IL-4/IL-13-responsive macrophages were linked to delayed resolution [96].

A second lipid-regulatory pathway in macrophages is controlled by sterol regulatory element-​binding proteins (SREBPs), which coordinate de novo lipogenesis [97]. In LPS-stimulated mouse macrophages, SREBP-1a supports lipogenesis and phagocytosis, and its deficiency impairs these responses [98]. SREBP-1a deficient mice also show defects in innate immune responses [99]. In IL-4-stimulated mouse macrophages, SREBP1-dependent fatty acid synthesis supports alternative activation [100]. These findings indicate that the functional consequences of SREBP activity depend on the activating stimulus and experimental setting.

Peroxisome proliferator activated receptor delta (PPARδ) participates in T lymphocyte metabolism (Fig. 3B). Memory CD8 positive T cells rely on FAO for long term persistence. PPARδ promotes a shift from glycolysis to oxidative metabolism and FAO while suppressing aerobic glycolysis during memory CD8^+^ T-cell formation, thereby supporting long-term cell survival [101, 102]. In CD, chronic inflammation may maintain T cells in persistent effector states. Activating PPARδ could redirect subsets toward memory or regulatory phenotypes by altering metabolic programs. PPARδ in mesenchymal stromal cells may also regulate Th17 responses through metabolic mechanisms [103]. Thus, lipid metabolism affects macrophages and T cells through distinct regulatory programs, with PPARγ and SREBPs shaping macrophage states and PPARδ influencing T-cell metabolic fate.

### Amino acid networks linking energy metabolism and epigenetics

**Fig. 4:**
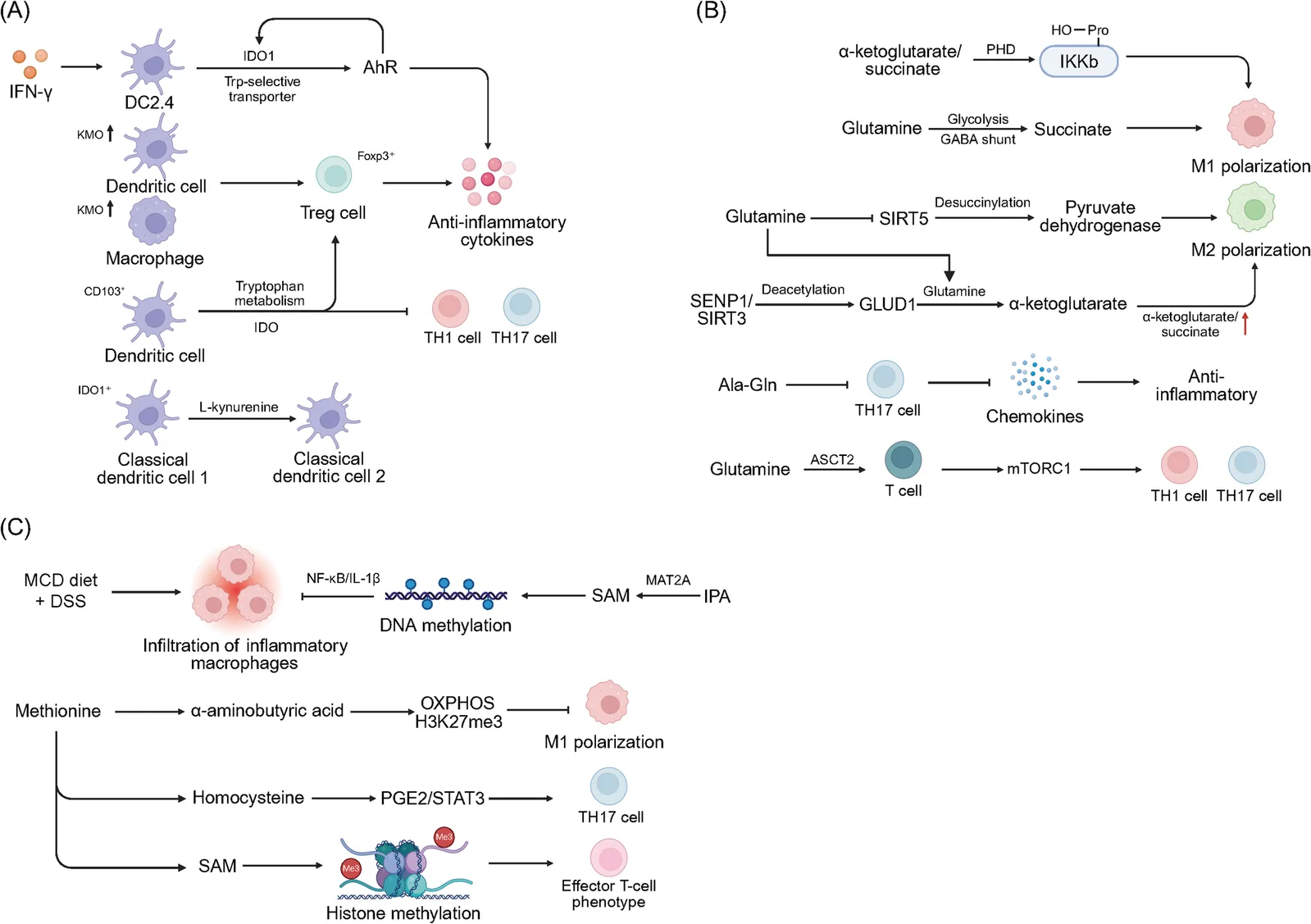
Amino Acid Metabolic Reprogramming of Macrophages and T Cells in IBD. (**A**) In TNBS colitis, colonic DCs and macrophages show increased KMO, while KMO deficiency attenuates inflammation with higher Foxp3^+^ Treg frequency and upregulated anti-inflammatory mediators, indicating DC/macrophage control of Treg generation via KMO metabolism and consequent impact on disease severity. In the gut, CD103^+^ DCs are the major IDO1^+^ population and, through Trp metabolism, induce Treg and restrain Th1/Th17. IDO1^+^ cDC1 extend immunoregulation to cDC2 by producing L-Kyn. IFN-γ induces the DC2.4 line to co-express IDO1 and a Trp-selective transporter, establishing a reinforcing Kyn–AhR loop. (**B**) Gln serves as a core substrate for immune-cell bioenergetics and metabolic reprogramming and directly modulates macrophage and T-cell function.In IL-4-stimulated mouse macrophages, Gln supports Arg1 and CD206 expression through regulation of SIRT5-mediated PDH desuccinylation. Succinate generated through glutaminolysis or the GABA shunt can support inflammatory macrophage activation. In macrophage activation models, a higher α-KG/succinate ratio favored expression of alternative-activation-associated genes, whereas a lower ratio favored pro-inflammatory readouts; In the macrophage activation model, α-KG-dependent PHD activity promoted IKKβ hydroxylation and restrained NF-κB signaling. In IL-4-stimulated mouse macrophages, SENP1–SIRT3 signaling deacetylates GLUD1, increases glutamine-derived α-KG production, and supports alternative activation. The dipeptide Ala-Gln suppresses Th17 generation, alleviates inflammation, and lowers chemokines in intestinal tissue, mitigating DSS colitis. Upon activation, naive T cells rapidly import Gln via ASCT2 to activate mTORC1, thereby promoting Th1/Th17 differentiation and inflammatory responses. (**C**) In mice with DSS-induced colitis, an MCD diet increases inflammatory macrophage infiltration and proinflammatory mediators. In mice with DSS-induced colitis, AABA increased OXPHOS and H3K27me3 and reduced macrophage-associated inflammatory readouts. The microbial metabolite IPA directly binds MAT2A, boosting SAM synthesis and DNA methylation to suppress NF-κB/IL-1β signaling. Hcy promotes Th17 differentiation and proliferation via the PGE2/STAT3 pathway. Activated T cells exhibit high demand for exogenous Met; raising intracellular SAM sustains histone methylation (e.g., H3K4me3) and supports effector programs. KMO, kynurenine 3-monooxygenase; IDO1, indoleamine 2,3-dioxygenase 1; cDC1/cDC2, conventional dendritic cell type 1/2; L-Kyn, L-kynurenine; AhR, aryl hydrocarbon receptor; SIRT5, sirtuin 5; PHD, prolyl hydroxylase; IKKβ, inhibitor of NF-κB kinase subunit β; SENP1, sentrin-specific protease 1; SIRT3, sirtuin 3; GLUD1, glutamate dehydrogenase 1; Ala-Gln, alanyl-glutamine; ASCT2, alanine–serine–cysteine transporter 2; AABA, α-aminobutyric acid; IPA, indole-3-propionic acid; MAT2A, methionine adenosyltransferase 2A; SAM, S-adenosylmethionine; Hcy, homocysteine; DC, dendritic cell; Trp, tryptophan; Gln, glutamine; GABA, gamma-aminobutyric acid; PDH, pyruvate dehydrogenase; α-KG, α-ketoglutarate; MCD, methionine- and choline-deficient; Met, methionine

Amino acids are central regulators of metabolism and are especially important in immunity and inflammation. Several specific amino acids have been linked to IBD. Tryptophan and its metabolites modulate inflammation, oxidative stress, immune responses, and intestinal homeostasis [104]. During IBD, macrophages often increase tryptophan uptake to support immunoregulatory and antimicrobial functions [105]. In trinitrobenzene sulfonic acid (TNBS)-induced colitis, kynurenine 3-monooxygenase (KMO) expression increased in colonic dendritic cells and macrophages. KMO deficiency attenuated inflammation and was accompanied by increased Foxp3 + Treg frequency and higher levels of anti-inflammatory mediator (Fig. 4A) [106]. In the intestinal immune environment, CD103 positive dendritic cells are major expressers of Indoleamine 2,3-dioxygenase 1 (IDO1). They induce Treg through tryptophan metabolism, limit T helper 1 and Th17 responses, and maintain oral tolerance and intestinal homeostasis [107]. IDO1 positive type 1 conventional dendritic cells (cDC1s) extend immunoregulatory activity to the type 2 conventional dendritic cells (cDC2s) subset by producing the tryptophan metabolite L-kynurenine [108]. IFN-γ induces IDO1 and the tryptophan selective transporter in the DC2.4 dendritic cell line, and this is reinforced through a kynurenine aryl hydrocarbon receptor (AhR) positive feedback loop [109]. Targeted metabolomics in two human IBD cohorts comprising 1069 patients identified alterations in tryptophan metabolism. In accompanying experimental models, supplementation with xanthurenic acid or kynurenic acid activated AhR, altered cellular energy metabolism, and reduced colitis severity. A recombinant aminoadipate aminotransferase intervention was also protective in rodent colitis [110].

Glutamine serves as a core substrate for energy and metabolic reprogramming in immune cells and directly regulates macrophage and T cell functions (Fig. 4B). Glutamine metabolism responds to environmental cues to shape macrophage phenotypes [111]. In IL-4-stimulated mouse macrophages, glutamine supports Arg1 and CD206 expression by suppressing SIRT5-mediated desuccinylation of pyruvate dehydrogenase [112]. Conversely, succinate generated through glutaminolysis or the gamma-aminobutyric acid shunt can support inflammatory macrophage activation in experimental models [113]. In macrophage activation models, a higher α-KG/succinate ratio favored expression of alternative-activation-associated genes, whereas a lower ratio favored pro-inflammatory readouts [114]. In the macrophage activation model, α-KG-dependent PHD activity promoted IKKβ hydroxylation and restrained NF-κB signaling [115]. In IL-4-stimulated mouse macrophages, the sentrin-specific protease 1/sirtuin 3 (SENP1/SIRT3) pathway deacetylates glutamate dehydrogenase 1, channels glutamine-derived carbon toward α-ketoglutarate, and supports alternative activation [116]. The alanyl-glutamine dipeptide suppresses Th17 generation, reduces chemokines, and mitigates weight loss and edema in DSS colitis [117]. Naive T cells require rapid glutamine uptake upon activation. Glutamine entry through alanine serine cysteine transporter 2 (ASCT2) drives mTORC1 activation and promotes Th1 and Th17 differentiation and inflammatory responses, whereas loss of this pathway weakens immunity [118].

Methionine metabolism has emerged as a hub in IBD immune regulation (Fig. 4C). In mice with DSS-induced colitis, a methionine- and choline-deficient diet worsens inflammation, increases colonic infiltration by inflammatory macrophages, and elevates proinflammatory mediators. Macrophage depletion partially alleviates disease, supporting a contribution of macrophages to the dietary exacerbation of DSS-induced inflammation [119]. Treatment with α-aminobutyric acid reduced macrophage-associated inflammatory readouts, with accompanying increases in OXPHOS and histone H3 lysine 27 trimethylation (H3K27me3) and a decrease in glycolysis [120]. The microbial metabolite indole-3-propionic acid binds methionine adenosyltransferase 2A, increases S-adenosylmethionine (SAM) production, and raises DNA methylation. This suppresses the NF-κB/IL-1β pathway and links macrophage inflammatory states to methionine metabolism and one carbon flux [121]. Homocysteine, a key intermediate of the methionine cycle, promotes Th17 differentiation and proliferation through the PGE2/STAT3 pathway and accelerates DSS colitis [122]. Activated T cells show high demand for exogenous methionine. Increasing intracellular SAM maintains histone methylation such as H3K4 trimethylation and supports effector programs, whereas limiting methionine supply reduces proliferation and responses [123]. Metabolomics in the T cell transfer colitis model shows altered levels of methionine and methionine sulfoxide, further linking methionine metabolism to intestinal inflammation [124]. Collectively, coupling among methionine, SAM, epigenetic regulation, and energy metabolism governs macrophage polarization and T cell differentiation and thereby shapes inflammatory intensity in IBD.

In sum, amino acid metabolism exerts major control over macrophage and T cell functions. Tryptophan, glutamine, and methionine act through distinct pathways to regulate inflammation and immune balance, and their alterations in patients and models closely track with disease activity.

## Epigenetic inscription of metabolic states in IBD

### Coupling of metabolic rewiring and chromatin control

**Fig. 5:**
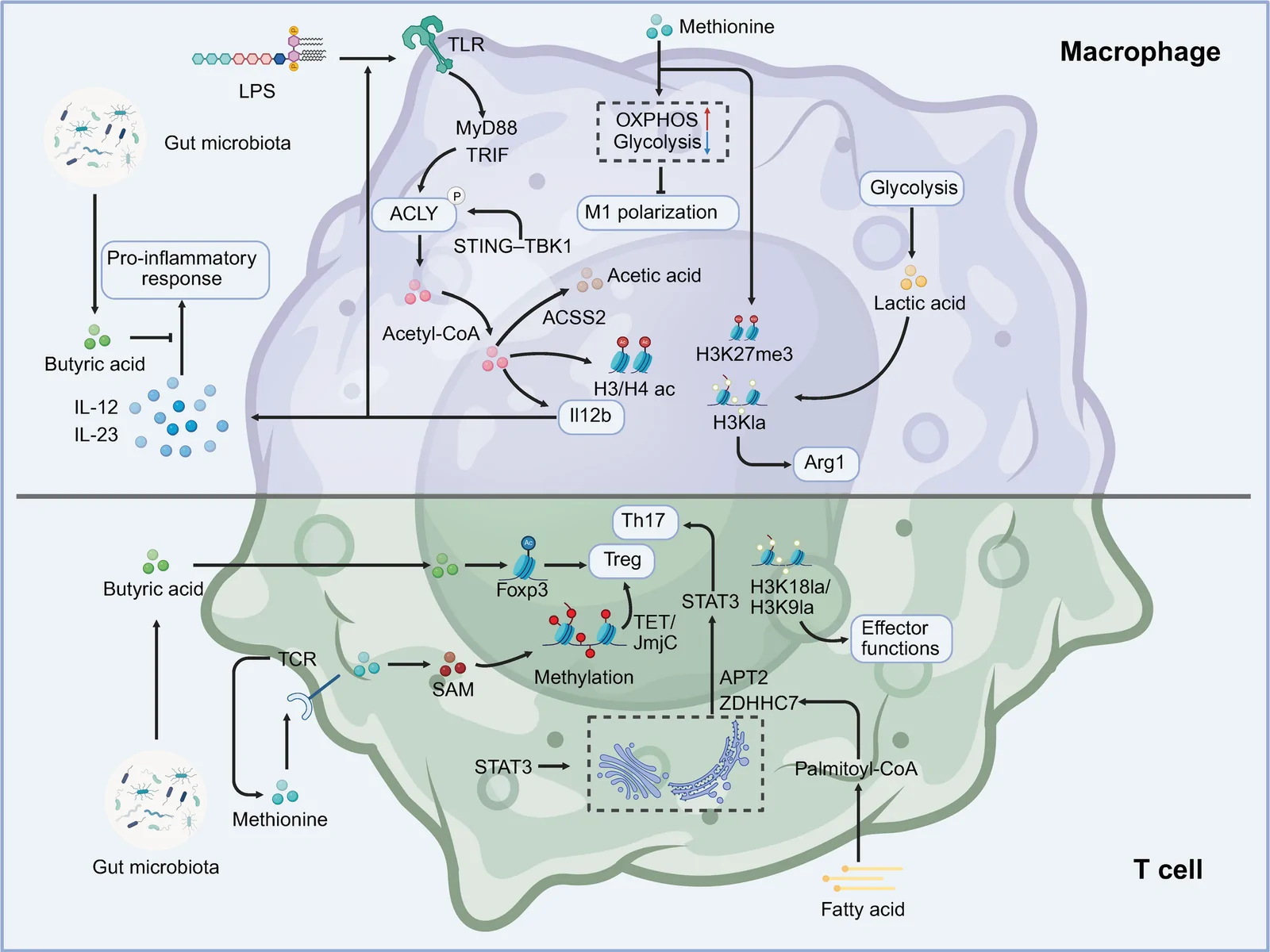
Metabolism and epigenetics coupling in immune cells. Acetylation: In macrophages, TLR–MyD88/TRIF can phosphorylate and activate ACLY, driving citrate carbon flux to acetyl-CoA, increasing H3/H4 acetylation and inducing LPS-responsive genes including *Il12b*; nuclear ACSS2 recycles acetate released by deacetylation to sustain acetylation and transcription. Butyrate from the gut microbiota acts as an endogenous HDAC inhibitor, reducing inflammatory responses in mouse intestinal lamina propria macrophages, and in mouse colonic T cells enhances H3 acetylation at the *Foxp3* locus to promote Treg differentiation. Methylation: Methionine flux determines intracellular SAM levels, thereby maintaining DNA and histone methylation and influencing Th lineages; TCR activation increases methionine uptake and methyl-donor generation. α-KG serves as a cofactor for TET/JmjC demethylases; lowering intracellular glutamine/α-KG promotes *FOXP3* promoter demethylation and induces a Treg-like phenotype, which is reversed by α-KG supplementation. The methionine-related metabolite AABA inhibits experimentally defined pro-inflammatory macrophage activation and alleviates DSS colitis, accompanied by increased OXPHOS, decreased glycolysis, and upregulated H3K27me3. Lactylation: Lactate produced by high glycolytic flux drives histone lactylation and directly regulates gene expression; increased H3Kla can coincide with Arg1 induction in experimental macrophage activation, but this association alone does not establish inflammation resolution; in CD8⁺ T cells, H3K18la/H3K9la couple to metabolic state to shape effector function. S-palmitoylation: Long-chain fatty acids serve as substrates for energy/membrane lipids and as donors for S-palmitoylation; the ZDHHC7/APT2-regulated STAT3 palmitoylation–depalmitoylation cycle determines its membrane localization, phosphorylation, and nuclear entry, promoting Th17 differentiation. Phosphorylation: In macrophages, TLR–MyD88/TRIF and STING–TBK1 can phosphorylate and activate ACLY, converting citrate carbon flux to acetyl-CoA. ACLY, ATP-citrate lyase; ACSS2, acyl-CoA synthetase short-chain family member 2; AABA, α-aminobutyric acid; Kla, lysine lactylation; JmjC, Jumonji C domain-containing; ZDHHC7, zinc finger DHHC-type palmitoyltransferase 7; APT2, acyl-protein thioesterase 2; TRIF, TIR-domain-containing adapter-inducing interferon-β; STING, stimulator of interferon genes; TBK1, TANK-binding kinase 1; DSS, dextran sulfate sodium; OXPHOS, oxidative phosphorylation

In the mucosal microenvironment of IBD, immune cells commonly display enhanced glycolysis together with local hypoxia. These metabolic cues are transduced into post-translational and epigenetic modifications that reprogram immune circuits and shape inflammatory phenotypes (Fig. 5).

For acetylation, glucose and acetate are converted to acetyl-CoA through ATP citrate lyase (ACLY) and acyl-CoA synthetase short-chain family member 2 (ACSS2). Acetyl-CoA directly fuels histone acetylation. In macrophages, TLR–MyD88/TRIF signaling phosphorylates and activates ACLY, diverting citrate carbon to acetyl-CoA, increasing H3 and H4 acetylation, and inducing LPS-responsive genes including *Il12b* [125]. Under hypoxia or nutrient limitation, nuclear ACSS2 recycles acetate released by deacetylation to sustain acetylation and transcription [126, 127].

For methylation and demethylation, methionine flux determines intracellular SAM levels to maintain DNA and histone methylation and influence T helper lineages. α-Ketoglutarate serves as a cofactor for ten-eleven translocation (TET) and Jumonji C (JmjC) demethylases. Exogenous α-ketoglutarate under Treg-polarizing conditions suppresses Foxp3^+^ Treg [128]. Conversely, lowering intracellular glutamine and α-ketoglutarate promotes demethylation at the *FOXP3* promoter and induces a Treg-like program, which is reversed by α-ketoglutarate supplementation [129].

For lactylation, high glycolytic flux generates lactate that drives histone lysine lactylation and directly regulates gene expression. In a mouse macrophage activation model, increased histone H3 lactylation coincided with later induction of Arg1 [130]. This association is context dependent and does not, by itself, establish inflammation resolution. In CD8^+^ T cells, H3K18 lactylation and H3K9 lactylation couple to metabolic state to sculpt effector function [131]. At the population level, genes related to lactylation are upregulated in inflamed CD tissue and correlate with immune infiltration [132].

Fatty-acid metabolism links to S-palmitoylation. Long-chain fatty acids serve as substrates for energy and membranes and as acyl donors for S-palmitoylation, thereby altering protein function and inflammatory thresholds. Dietary long-chain fatty acids promote epithelial STAT3 palmitoylation through CD36, amplifying inflammation and disrupting the barrier [133].

Phosphorylation couples receptor inputs to metabolic pathways. In macrophages, TLR–MyD88/TRIF and STING–TBK1 phosphorylate and activate ACLY, redirecting citrate to acetyl-CoA and rewiring metabolism at its source [125, 134].

### Histone acetylation in mucosal immunity

**Fig. 6:**
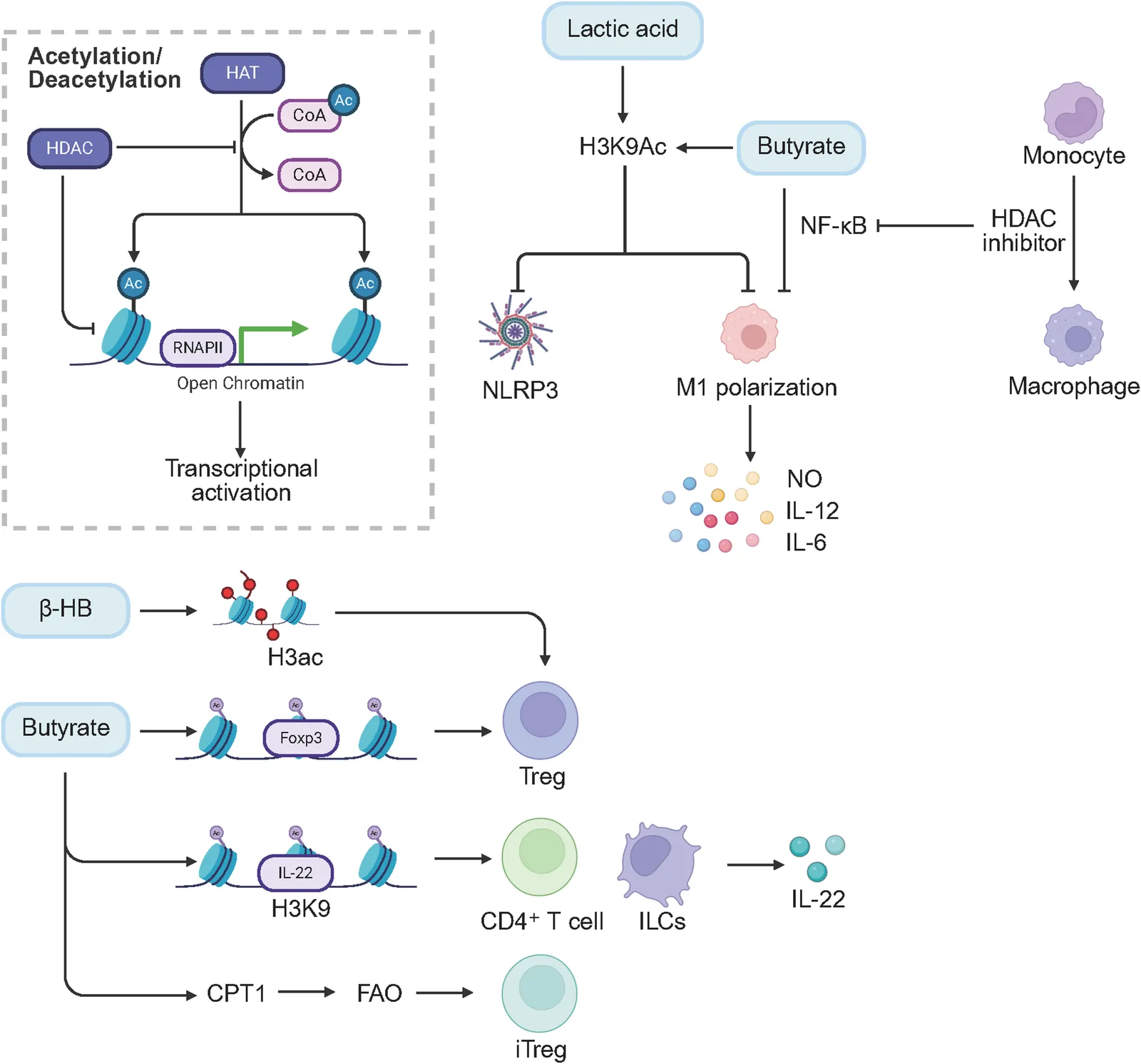
Epigenetic control of gut inflammation via histone acetylation in macrophages and T cells. Histone acetyltransferases (HATs) add acetyl groups to histone lysine residues, whereas histone deacetylases (HDACs) remove them. These modifications influence chromatin accessibility and transcription, with effects that depend on the genomic locus and responding cell type. HDAC inhibitors can reduce disease severity in experimental colitis by modulating inflammatory or regulatory responses. In mouse BMDMs, lactic acid increased H3K9Ac and H3K18la and was associated with reduced iNOS/CD86-associated inflammatory readouts and NLRP3-related pyroptosis; protective effects were also observed in DSS-induced colitis mice. Butyrate increases H3K9 acetylation and reduces LPS-induced NO production and IL-12 and IL-6 expression in mouse BMDMs and LP macrophages; it also blocks NF-κB signaling to exert anti-inflammatory effects. Butyrate promotes H3 acetylation across Foxp3 CNS and promoter regions in naive T cells, inducing Treg differentiation. It also enhances H3K9 acetylation at the IL-22 promoter HRE, boosting IL-22 production by CD4^+^ T cells and ILCs. In addition, butyrate increases CPT1-mediated FAO to promote iTreg generation and inhibits HDAC3 to restrain Th17 differentiation. Beyond butyrate, β-HB similarly elevates H3 acetylation, promotes Treg, lowers the colonic Th17/Treg ratio, and augments Foxp3 expression, thereby improving colitis. H3K9Ac, histone H3 lysine 9 acetylation; NLRP3, NLR family pyrin domain–containing 3; HDACi, histone deacetylase inhibitor; BMDM, bone marrow–derived macrophage; LP, lamina propria; CNS, conserved noncoding sequence; HRE, hypoxia response element; ILC, innate lymphoid cell; CPT1, carnitine palmitoyltransferase 1; FAO, fatty acid oxidation; iTreg, induced regulatory T cell; β-HB, β-hydroxybutyrate

Histone acetylation is regulated by histone acetyltransferases (HATs), which add acetyl groups to lysine residues, and histone deacetylases (HDACs), which remove them. These activities influence chromatin accessibility and transcription (Fig. 6) [135]. Their effects depend on the genomic locus and responding cell; increased acetylation is not uniformly anti-inflammatory [136]. Experimental modulators and their model-specific effects are summarized in Table 1.

**Table 1:** Experimental modulators of immune-cell modifications in IBD: acetylation, methylation, lactylation, palmitoylation and phosphorylation

Modification	Intervention	Target	Model	Effect	References
Histone acetylation	Lactate	Promotes H3K9 acetylation	Mice with DSS-induced colitis, bone marrow–derived macrophages	Reduced iNOS/CD86-associated inflammatory readouts and NLRP3-related pyroptosis; attenuated DSS-induced colitis.	[139]
	ESM-HDAC528	HDAC (targeted to CES1^+^ monocytes/macrophages)	human CES1 transgenic mice, human CD14^+^ monocytes/macrophages	Reduces monocyte-macrophage differentiation and IL-6/TNF-α	[140]
	Butyrate	-	THP-1 cells, TNBS-treated rats	Inhibits p65 nuclear translocation; lowers pro-inflammatory mediators; ameliorates TNBS colitis	[141]
	Sodium butyrate	HDAC; inhibits NF-κB signaling	RAW264.7 cells, COLO205 cells, peritoneal macrophages, DSS-treated mice; Il10^−/−^ mice	Blocks IκBα degradation/phosphorylation and NF-κB binding; improves acute/chronic colitis	[142]
	NMJ-2	HDAC	RAW264.7 cells, Wistar rats	Suppresses LPS-induced NF-κB phosphorylation/expression; mitigates colitis pathology	[262]
	CKD-506	HDAC6	RAW264.7 cells, DSS-treated mice	Inhibits IκBα phosphorylation/degradation and NF-κB DNA binding; alleviates acute and chronic colitis	[143]
	Butyrate	HDAC; increases H3K9Ac at HREs in the IL-22 promoter	CD4^+^ T cells, mouse colitis models	Enhances IL-22 secretion; suppresses colitis	[144]
	Faecalibacterium prausnitzii	HDAC3	Th17 cells, mouse colitis models	Suppresses Th17 differentiation/IL-17; alleviates colitis	[146]
	Faecalibacterium prausnitzii	HDAC1	Th17 cells, mouse colitis models	Maintains Th17/Treg balance; ameliorates colitis	[147]
	β-Hydroxybutyrate	Increases histone H3 acetylation	Mouse colitis models	Increases Treg frequency, reduces the Th17/Treg ratio, elevates Foxp3 expression; ameliorates colitis	[148]
	RGFP109	HDAC1/HDAC3	Human PBMCs cell, mouse colitis models	Inhibits Th17 differentiation and IL-17A; eases colitis	[149]
	Tubacin	HDAC6	Colonic Foxp3^+^ Tregs cells, DSS-treated colitis models	Enhances Treg suppressive function; protective in DSS colitis	[150]
	HDAC9 deficiency	HDAC9	*Hdac9* ^-/-^ mice	Augments Treg function and prevents colitis	[151]
	Trichostatin A	HDAC	DSS mice	Increases Foxp3 acetylation and Foxp3^+^ Tregs; reduces DSS colitis severity	[263]
Methylation	IGF2BP2 deficiency (genetic)	IGF2BP2	BMDMs, mice with DSS-induced colitis	IGF2BP2 deficiency enhanced LPS-responsive inflammatory activation in BMDMs and aggravated DSS-induced colitis.	[153]
	DNA methylation inhibition	DNMTs	THP-1-derived macrophages, mouse obesity model	Altered macrophage marker expression toward an anti-inflammatory profile in THP-1 cells and an obesity mouse model.	[157]
	Bacteroides fragilis toxin	METTL3	IBD patient macrophages; mouse infection model	Reduces m^6^A via METTL3 repression, stabilizes ITGA5 mRNA, and promotes macrophage activation	[154]
	HNRNPC knockdown	HNRNPC	LPS-stimulated macrophages, mice with DSS-induced colitis	Dampens inflammatory activation; regulates *Itgb7* mRNA splicing/stability to curb migration	[155]
	Coptisine	METTL14	Macrophages, mice with DSS-induced colitis	Increased METTL14-mediated m6A modification and TSC1 mRNA stability; modulated macrophage activation and attenuated DSS-induced colitis.	[156]
	UHRF1 deletion / mutation	UHRF1	Macrophages, Macrophage specific knockout mice	Hypomethylates Tnf-α promoter, increases TNF-α, and worsens colitis	[158]
	SETD8 knockdown or inhibition (UNC0379)	SETD8	RAW264.7 macrophages, mice with DSS-induced colitis	Reduces H4K20me1 at p62 promoter, elevates inflammatory mediators; inhibition aggravates colitis	[159]
	JMJD3/KDM6B activity	JMJD3	Mice with DSS-induced colitis, macrophages	Removes H3K27me3 at Nrf2 promoter to drive NLRP3 inflammasome activation; aggravates colitis (inhibition ameliorates)	[160]
	SETD2 in Treg cells	SETD2	Mouse Treg cells; mice with Treg-specific Setd2 deficiency	Maintains GATA3^+^ ST2^+^ tTreg programs and intestinal homeostasis; loss skews toward Th2 and intestinal inflammation	[161]
	SETD2 in T cells	SETD2	CD4^+^T cells, mouse EAE model	Catalyzes H3K36me3 at *Lpcat4* to boost phosphatidylcholine, limit ER/oxidative stress, restrain Th17 and promote iTreg	[162]
	AMI-1, PRMT5 knockdown	PRMT5	Human CD4^+^ T cells from patients with UC, mice with DSS-induced colitis	Increases Foxp3^+^ Treg differentiation and function; lowers TNF-α/IL-6/IL-13; attenuates colitis	[163]
	Selective EZH2 inhibition	EZH2	Mouse models of intestinal inflammation	Alleviates intestinal inflammation; promotes MDSC-mediated immunosuppression with minimal effect on Treg stability	[165]
	JMJD3 in effector T cells	JMJD3	T cells	Demethylates H3K27 at *Rorc* and Th17 cytokine loci, stabilizing RORγt and promoting IL-17/IL-22 secretion	[166]
Lactylation	lactate-producing Saccharomyces cerevisiae	Increases H3K18 lactylation	Mice with DSS-induced colitis, BMDMs	Suppresses NLRP3 inflammasome activation and macrophage pyroptosis; attenuates colitis	[139]
	BCAP	Promotes histone lactylation	bone marrow derived macrophages	Restores Arg1 and Klf4; drives the transition from inflammatory to reparative macrophages; required for DSS-colitis recovery	[182]
	Gegen Qinlian decoction	Reduces pan-Kla and H3K18la	Mice with DSS-induced colitis	Reduced macrophage inflammatory readouts and attenuated experimental colitis, alongside reduced histone lactylation.	[185]
Palmitoylation	ZDHHC17	NLRP3 Cys419	THP-1 cells; BMDMs; mouse DSS colitis	Enhances NLRP3–NEK7 interaction and inflammasome activation; inhibition lowers IL-1β/ASC and alleviates DSS colitis	[190]
	ZDHHC5	NLRP3 Cys837/Cys838 (human); Cys834/Cys835 (mouse)	THP-1 cells, *Zdhhc5*^−/−^ mice	Promotes NLRP3 oligomerization, NLRP3–NEK7 interaction, ASC aggregation, caspase-1 activation, and IL-1β/IL-18 release	[191]
	ZDHHC7	NLRP3 Cys126	BMDMs, THP-1 cells	Controls NLRP3 localization at TGN/dTGN, promotes ASC recruitment/oligomerization, and drives inflammasome activation	[192]
	ZDHHC12	NLRP3 Cys841	THP-1 cells, mouse inflammatory models	Terminates inflammasome signaling by promoting NLRP3 degradation via chaperone-mediated autophagy; deficiency exacerbates inflammation	[193]
	2-Bromopalmitate	Global palmitoylation	THP-1 cells, mice with DSS-induced colitis	Reduces NLRP3 palmitoylation/activation and IL-1β secretion; improves weight loss, survival, and histology in DSS colitis	[190]
	ABHD17A	NLRP3	THP-1 cells	Depalmitoylates NLRP3; knockdown increases NLRP3 palmitoylation and inflammasome activation	[191]
Phosphorylation	Fostamatinib R788	SYK	UC patient tissue; mouse DSS colitis; mouse macrophages	Reduced macrophage ROS and pro-inflammatory readouts, restored phagocytosis and bactericidal activity, and attenuated experimental colitis.	[197]
	SYK S544Y knock-in	SYK with increased autophosphorylation	SYK S544Y knock-in mice	Increases SYK, NF-κB and MAPK activation; enhances macrophage infiltration; worsens colitis	[198]
	Mincle blockade	Mincle–SYK axis	CD patient samples, mouse colitis, macrophages	Limits macrophage pyroptosis and pro-inflammatory cytokines; reduces neutrophil chemotaxis; improves colitis	[199, 200]
	CCL4-targeted nanoparticles delivering a SYK inhibitor	SYK	Mouse colitis models	Improves intestinal barrier function and mitigates colitis by targeted delivery of SYK inhibition	[201]
	Macrophage-specific p38α deletion / p38 inhibitors	MAPK p38α	Human colitis tissue, mouse DSS colitis, macrophages	Reduces inflammatory mediators and supports epithelial repair; alleviates colitis	[202]
	SHP2 deletion or inhibition	SHP2	IBD patient tissues, mouse colitis, macrophages	Restores IL-10–STAT3 anti-inflammatory signaling in macrophages and attenuates intestinal inflammation	[203]
	Selective JAK1 inhibitor and tofacitinib	JAK1 / JAK–STAT	Human monocyte-derived macrophages, mouse acute DSS colitis	Induces anti-inflammatory macrophage phenotype; modest protection in acute rescue colitis	[204]
	Pak2 phosphorylation of RORγt	Pak2 phosphorylates RORγt at S316	Mouse T cells, adoptive-transfer colitis	Pak2-dependent phosphorylation promotes Itch-mediated RORγt degradation; limits IL-17 and protects from colitis	[205]
	Porphyromonas gingivalis exposure	STAT1 Ser727 in the AHR–STAT1 axis	Mouse colitis	Decreases STAT1 Ser727 phosphorylation; impairs Treg differentiation and promotes Th17 responses; aggravates colitis	[206]
	USP28 deficiency	STAT5 signaling	Mouse T cells, mice with DSS-induced colitis	Increases STAT5 phosphorylation and iTreg features with reduced IL-17; decreases IL-22 and increases susceptibility to DSS colitis	[207]
	RGS10 deficiency	STAT1 and STAT3 phosphorylation in CD4^+^ T cells	UC patient samples; *Rgs10*^−/−^ mice	Reduces STAT1/STAT3 phosphorylation in CD4^+^ T cells; suppresses Th1/Th17 differentiation; alleviates colitis	[208]
	Honokiol	SIRT3 regulation of the STAT3 and RORγt pathway	UC patient samples, mouse colitis, T cells	Activates SIRT3; inhibits STAT3 phosphorylation and RORγt; reduces Th17 differentiation; ameliorates colitis	[209]
	Ethanol exposure	TRPV1 activates MAPK ERK p38 and NF-κB	Mouse colitis, macrophages	Increases macrophage TNF, IL-1β, and IL-10 production and exacerbates experimental colitis	[138]

Macrophage inflammatory responses and their regulation contribute to IBD pathogenesis and mucosal homeostasis [137, 138]. HDAC inhibitors can attenuate experimental colitis by modulating these responses. In LPS-stimulated mouse BMDMs, lactic acid increased H3K9 acetylation and H3K18 lactylation. It was also associated with reduced iNOS/CD86-associated inflammatory readouts and NLRP3-related pyroptosis. In DSS-treated mice, lactic acid or lactic acid-producing Saccharomyces cerevisiae attenuated colitis and increased colonic CD206-associated macrophage readouts [139]. The esterase-sensitive HDAC inhibitor ESM-HDAC528 reduces differentiation of colonic monocytes into macrophages and dampens DSS-induced macrophage activation, with therapeutic benefit in T cell transfer colitis [140]. Butyrate likewise regulates macrophage function through epigenetic mechanisms. Butyrate increases H3K9 acetylation and reduces LPS-induced nitric oxide production and IL-12 and IL-6 expression in mouse bone marrow-derived and intestinal lamina propria macrophages [15]. Butyrate also blocks NF-κB signaling by inhibiting LPS-induced nuclear translocation of p65 in THP-1 cells and peripheral blood mononuclear cells (PBMCs) and is validated in 2,4,6-trinitrobenzenesulfonic acid (TNBS) colitis [141]. In RAW264.7 macrophages, butyrate reduces NF-κB binding and prevents IκBα degradation and phosphorylation, improving histology in colitis models [142]. The HDAC6 inhibitor CKD-506 suppresses NF-κB in macrophages and intestinal epithelial cells and is effective in acute and chronic mouse colitis [143].

Beyond macrophages, butyrate promotes acetylation across conserved noncoding sequences and the promoter of Foxp3 in naive T cells to induce Treg and alleviate T cell-dependent colitis [14]. Butyrate enhances acetylation of H3K9 at hypoxia-response elements within the IL-22 promoter and increases IL-22 secretion by CD4^+^ T cells and innate lymphoid cells to suppress colitis [144]. It also raises CPT1-mediated FAO and promotes induced Treg generation to attenuate DSS colitis [145]. Butyrate inhibits Th17 differentiation through HDAC3 and improves colitis in animals [146]. It blocks the IL-6–STAT3–IL-17 pathway through HDAC1 inhibition and promotes Foxp3 expression to maintain the Th17/Treg balance and reduce colorectal inflammation [147]. β-Hydroxybutyrate similarly increases histone H3 acetylation, promotes Treg differentiation, lowers the Th17/Treg ratio, and boosts Foxp3 expression to ameliorate colitis [148]. Pharmacologically, the selective HDAC1/3 inhibitor RGFP109 suppresses Th17 differentiation and IL-17A secretion by PBMCs and eases colitis [149]. The HDAC6 inhibitor tubacin enhances suppressive function of colonic Foxp3^+^ Treg through inhibition of heat-shock protein 90 and protects in DSS colitis [150]. HDAC9 deficiency augments Treg function and heat-shock protein 70 and prevents colitis in mice [151].

### DNA, RNA and histone methylation in inflammatory regulation

Methylation has emerged as a key epigenetic mechanism in inflammation [152]. In mouse BMDMs, deficiency of the N6-methyladenosine (m6A) reader insulin-like growth factor 2 mRNA-binding protein 2 (IGF2BP2) enhances LPS-responsive inflammatory activation and impairs IL-4-induced activation; myeloid IGF2BP2 deficiency also aggravates DSS-induced colitis [153]. In IBD and in mice with high-level enterotoxigenic Bacteroides fragilis infection, Bacteroides fragilis toxin represses FOXD3 to downregulate methyltransferase-like 3 (METTL3) transcription, reducing m^6^A in inflammatory macrophages. Loss of m^6^A stabilizes ITGA5 mRNA and increases its expression, which promotes macrophage activation through integrin-dependent signaling [154]. Under inflammatory conditions, the m^6^A reader heterogeneous nuclear ribonucleoprotein C (HNRNPC) rises in colitic macrophages. Knockdown alleviates LPS-induced activation. Transcriptomics indicate that it constrains alternative splicing and regulates stability of *Itgb7* mRNA to control inflammatory responses and macrophage migration [155]. Other methyltransferases are also involved. In mice with DSS-induced colitis, coptisine upregulates methyltransferase-like 14 (METTL14), enhances m6A modification, and stabilizes TSC1 mRNA to modulate macrophage activation and attenuate experimental colitis [156].

DNA methylation is likewise critical in macrophages. In an obesity-related study, inhibition of DNA methylation altered macrophage marker expression toward an anti-inflammatory profile; this observation was not established directly in human IBD [157]. Uhrf1 deletion or mutation of its DNA-methylation control sites exacerbates DSS colitis with hypomethylation at the *Tnf* promoter and increased TNF-α expression, indicating a role for Uhrf1-mediated DNA methylation in restraining inflammatory cytokines [158].

Histone methylation also shapes inflammation. In IBD, p62 is upregulated under histone control. In colitis, the H4K20 methyltransferase SETD8 is downregulated. Knockdown in LPS-activated macrophages increases multiple inflammatory mediators. Mechanistically, SETD8 loss reduces H4K20me1 at the p62 promoter, and silencing p62 partially reverses the proinflammatory effect [159]. The demethylase Jmjd3 removes repressive H3K27me3 at the Nrf2 promoter to enhance NRF2 expression, which drives NLRP3 inflammasome activation and macrophage inflammatory function [160].

Treg function is tightly linked to transcriptional programs controlled by methylation. The H3K36 methyltransferase Setd2 promotes expression and cooperation of GATA3 and ST2. In mice with Treg-specific Setd2 deficiency, IL-33 favors Th2 expansion rather than activation of GATA3^+^ Treg cells, indicating that Setd2 supports Treg-mediated suppression of Th2 responses and maintenance of immune homeostasis [161]. Setd2 also catalyzes H3K36me3 at the *Lpcat4* promoter to increase *Lpcat4* expression. The resulting phosphatidylcholine alleviates endoplasmic reticulum and oxidative stress and suppresses HIF-1α to limit Th17 differentiation and promote Treg development [162]. In colitis and UC, protein arginine methyltransferases (PRMTs) are elevated. PRMT5 increases H3K27me3 and DNMT1 binding at the *Foxp3* promoter to raise promoter methylation, restrict Treg differentiation, and elevate TNF-α, IL-6, and IL-13. Lowering PRMT5 increases Treg and reduces inflammation with protection from colitis [163]. Enhancer of zeste homolog 2 (EZH2) maintains FOXP3-driven Treg stability and suppressive function through H3K27me3. Loss destabilizes this barrier, converts Treg into proinflammatory effectors, and aberrantly activates Th1 and Th17 programs to worsen autoimmunity and intestinal inflammation [164]. By contrast, selective inhibition of this methyltransferase activity promotes myeloid-derived suppressor cells generation and an immunosuppressive milieu to reduce tissue-damage-induced inflammation without markedly altering Treg [165]. In effector T cells, Jmjd3 removes H3K27me3 at *Rorc* and Th17 cytokine loci to stabilize RORγt expression and IL-17/IL-22 secretion, driving Th17 differentiation and inflammation [166].

### Lactylation as a metabolic signal to chromatin

Lactate is not only an end product of glycolysis but also a signaling metabolite in immune regulation [167]. Intracellular accumulation of lactate alters histone lactylation in a dose-dependent fashion [168]. Glycolysis inhibitors reduce lactylation, whereas glucose supplementation or mitochondrial inhibitors increase lactate and promote lactylation [169]. Histone lactylation is a post-translational modification driven by lactate whose steady state reflects the combined action of writers, erasers, and readers, analogous to methylation and acetylation [170]. The acetyltransferase p300 can serve as a lactyltransferase that uses lactyl-CoA as substrate to catalyze lysine lactylation. Overexpression or inhibition of p300 markedly shifts lactylation levels [171, 172]. HDAC1 and HDAC3 act as delactylases at defined sites, and loss of HDAC3 increases H4K5 lactylation [173]. Brg1 binds H3K18 lactylation and accumulates at mesenchymal-to-epithelial transition (MET)-related promoters as a reader [174]. Histone lactylation enhances transcription of inflammation-related genes [175].

H3K18 lactylation stabilizes the YTHDF2/Kcnk6 complex, activates the NLRP3 inflammasome with a 2.3-fold increase in apoptosis-associated speck-like protein containing a CARD (ASC) oligomers, and promotes IL-1β secretion [176]. In CD, gastrointestinal lactate levels are increased [177]. Macrophages display high histone lactylation with reduced SIRT1 expression. These changes inversely correlate with OXPHOS capacity and infiltration by proinflammatory populations such as Th17. SLC16A1-mediated monocarboxylate transport regulates these processes [178]. In UC, lactate-mediated immunoregulation is also prominent. Lactate can act through the cell-surface receptor G-protein-coupled receptor 81 (GPR81) to regulate intestinal immune responses and protect mice from experimental colitis [179]. In inflammatory macrophage models, lactylation of PKM2 at lysine 62 (K62) stabilizes its tetrameric form and restrains inflammatory metabolic adaptation [180]. Exogenous lactate has also been reported to suppress TLR4–NF-κB signaling and reduce inflammatory macrophage activation in experimental colitis [181].

In the lactate-producing yeast study, monocarboxylate transporter 1 (MCT1), encoded by SLC16A1, was implicated in macrophage responses to lactate. Lactic acid restored H3K9 acetylation and H3K18 lactylation relative to LPS treatment and was associated with reduced NLRP3-related pyroptosis. A direct, locus-specific causal link between these histone changes and NLRP3 transcription was not established [139]. The B-cell adaptor for phosphoinositide 3-kinase (BCAP) links macrophage metabolism to repair-associated histone lactylation. BCAP-deficient macrophages show reduced Arg1 and Klf4 expression. Under conditions of increased histone lactylation, these bone marrow-derived macrophages restore Arg1 expression within 12 h, accompanied by increased Klf4 expression. In DSS colitis, BCAP loss blocks the inactivation of FOXO1 and GSK3β, reduces aerobic glycolysis and lactate production, lowers histone lactylation, and suppresses repair-associated genes, thereby worsening disease [182]. In LPS-stimulated macrophages, histone lactylation correlates with Arg1 expression but is uncoupled from the expression of genes associated with resolving macrophage activation; LPS-induced Arg1 expression depends on IL-6–STAT3 signaling [183]. Mitochondrial dynamics intersect with lactylation. In mouse bone marrow-derived macrophages, IL-4-stimulated cells exhibit more mitochondrial fragmentation than cells stimulated with LPS and IFN-γ [184]. Loss of fusion raises basal Arg1. In this experimental setting, mitochondrial fragmentation is associated with lactate accumulation, increased histone lactylation, and resolution-associated responses [184]. In a DSS-induced colitis study, Gegen Qinlian decoction attenuated inflammatory readouts alongside reduced pan-lysine lactylation and H3K18 lactylation [185]. These findings support a context-dependent interpretation rather than a uniformly beneficial effect of increased lactylation.

Lactate and histone lactylation also shape T-cell function. H3K18 and H3K9 lactylation mark active promoters and enhancers that govern CD8^+^ T-cell states [131]. H3K9 lactylation correlates with mitochondrial fusion in naive and memory CD8^+^ T cells, whereas H3K18 lactylation promotes mitochondrial fission in activated cells and supports heightened glycolysis [186]. Exogenous lactate does not further increase these marks in already activated CD8^+^ T cells but markedly increases lactylation in naive and memory cells, indicating state-specific effects [186]. Inhibition of LDHA reduces lactylation and impairs CD8^+^ T-cell effector function [186]. Bulk and single-cell transcriptomic analyses identify lactylation-associated gene signatures in inflamed CD tissue; these analyses do not directly measure protein lactylation [132]. SLC16A1-mediated lactate uptake supports the suppressive function of tumour-infiltrating Treg cells [187]. In CD, SLC16A1 negatively correlates with TCR signaling and positively with Th17 infiltration [132]. Embigin promotes MCT localization to the plasma membrane [188] and is reduced in CD with inverse correlations to epithelial bacterial invasion and Th17 infiltration [132]. Lactate also reprograms Th17 plasticity by suppressing IL-17A and inducing Foxp3 with increased H3K18 lactylation. High lactate in inflamed intestine reduces Th17 pathogenicity and can convert Th17 toward regulatory phenotypes in vivo [189].

### Palmitoylation as a lipid switch for inflammasomes and T-cell signaling

In macrophages, NLRP3 palmitoylation at Cys419 is catalyzed by ZDHHC17 and strengthens interaction with NIMA-related kinase 7 (NEK7) to drive inflammasome activation. Inhibiting this modification reduces IL-1β secretion and ASC aggregation and improves disease in mouse IBD models [190]. ZDHHC5 catalyzes S-palmitoylation at Cys834/Cys835 in mouse NLRP3, corresponding to Cys837/Cys838 in human NLRP3, to promote inflammasome activation [191]. ZDHHC7 modifies Cys126 in mouse NLRP3, corresponding to Cys130 in human NLRP3, to regulate its localization at the trans-Golgi network and dispersed trans-Golgi network, promoting ASC polymerization and inflammasome activation [192]. By contrast, ZDHHC12 palmitoylates Cys841 in mouse NLRP3, corresponding to Cys844 in human NLRP3, to negatively regulate inflammasome signaling [193]. Mechanistically, ZDHHC7-driven Cys126 palmitoylation is present at rest and persists after activation to anchor NLRP3 before transport toward the microtubule-organizing center. Subsequently, ZDHHC5 at Cys834/Cys835 and ZDHHC17 at Cys419 enhance NLRP3–NEK7 interaction. In the late phase, ZDHHC12 triggers Cys841 palmitoylation to terminate signaling. Thus site-specific S-palmitoylation of NLRP3 is temporally ordered and functionally distinct [192, 194].

In T cells, ZDHHC7 and ZDHHC3 palmitoylate STAT3 at Cys108 to promote plasma-membrane localization together with IL6ST and JAK2, enabling STAT3 phosphorylation. Phosphorylated STAT3 then undergoes acyl-protein thioesterase 2 (APT2)-mediated depalmitoylation to enter the nucleus and induce genes essential for Th17 differentiation. Either blocking palmitoylation by deleting ZDHHC7 or inhibiting APT2-mediated depalmitoylation suppresses STAT3 signaling and protects in mouse colitis [195]. Evidence linking NLRP3 and STAT3 palmitoylation to intestinal inflammation comes mainly from murine models and cultured cells [190, 195]. These mechanisms require direct validation in macrophages and T cells from human IBD mucosa.

### Phosphorylation nodes integrating receptor input and metabolic status

Spleen-associated tyrosine kinase (SYK) is a central kinase in immune signaling. Upon receptor activation, it is recruited and activated and then propagates signals through PI3K/AKT, RAS/ERK, and NF-κB to control proliferation, differentiation, and cytokine production [196]. SYK expression is increased in lamina propria macrophages in UC. In the accompanying experimental models, SYK activity promotes reactive oxygen species (ROS) production and inflammatory macrophage responses, whereas inhibition with R788 improves phagocytic and bactericidal functions and reduces inflammatory readouts [197]. Inhibitors such as R788 restore phagocytic and bactericidal functions, reduce ROS, and limit M1 polarization [197]. Gain-of-function variants increase autophosphorylation and activate downstream NF-κB and mitogen-activated protein kinase (MAPK) pathways to cause immune dysregulation [198]. In acute colitis, S544Y knock-in mice show worse disease with enhanced NF-κB and MAPK pathways activation and increased macrophage infiltration, indicating promotion of colitis via macrophage functional changes [198]. Gain-of-function SYK variants can dysregulate macrophage podosome formation [199]. During intestinal inflammation, activation of macrophage Mincle–SYK signaling promotes NLRP3-mediated pyroptosis and proinflammatory cytokine release [199, 200]. Macrophage SYK activation is also linked to epithelial myosin light-chain kinase (MLCK) signaling and myosin light-chain phosphorylation (p-MLC), which promote tight-junction remodeling and barrier injury [201].

p38α is another key MAPK. It is elevated in colitic macrophages, and its deletion alleviates DSS colitis by reducing inflammatory mediators and enhancing epithelial repair, in part by regulating *Nlrp1b* and Oas family genes [202]. Hyperactivation of MAPK/p38, MAPK/ERK, and NF-κB can also induce a macrophage cytokine profile characterized by increased TNF, IL-1β, and IL-10 production and exacerbate experimental colitis [138].

SH2 domain-containing protein tyrosine phosphatase 2 (SHP2) expression is increased in colonic macrophages and blood monocytes in IBD. SHP2 signaling disrupts the macrophage IL-10–STAT3 anti-inflammatory axis and aggravates disease [203]. In human monocyte-derived macrophages, tofacitinib and a selective JAK1 inhibitor suppress STAT1 phosphorylation and IFN-γ-induced inflammatory responses [204].

In T cells, phosphorylation programs set the Th17/Treg balance. Pak2-mediated phosphorylation of RORγt promotes conformational change and ubiquitin-mediated degradation to limit IL-17. Loss of this phosphorylation increases Th17 pathogenicity and worsens colitis [205]. Porphyromonas gingivalis reduces microbial linoleic-acid metabolism and weakens AHR ligands, which lowers STAT1-Ser727 phosphorylation, suppresses Treg differentiation, and enhances Th17 differentiation [206]. In Usp28-deficient mice, STAT5 phosphorylation increases in activated T cells, with reduced IL-17 production, enhanced induced Treg (iTreg) features, and decreased IL-22 production, alongside greater susceptibility to DSS-induced colitis [207]. RGS10 promotes STAT1 and STAT3 phosphorylation in CD4^+^ T cells to drive Th1/Th17 differentiation and worsen inflammation; its loss blocks these events and eases colitis [208]. Activating SIRT3 inhibits STAT3/RORγt signaling to limit Th17 differentiation with protection in colitis [209].

## Inflammatory memory and relapse-prone immune states in IBD

During IBD remission, mucus-layer homeostasis and immune activity are only partially restored [210]. In the colonic mucosa of patients with UC in clinical and endoscopic remission, the immune molecular profiles related to T helper cells and Treg remain persistently shifted from those of healthy controls [211]. In a mouse dextran sodium sulfate (DSS) model of barrier injury, translocation of the intestinal microbiota to the bone marrow reprograms myeloid progenitors through Mincle, such that the macrophages subsequently derived from these progenitors display stronger inflammatory responses upon restimulation and aggravate colitis [212]. Chromatin studies in mouse bone marrow derived macrophages and intestinal macrophages further showed that bacterial exposure together with IL-10 deficiency jointly remodels chromatin accessibility at inflammation related loci, indicating that in the IBD setting macrophages do not merely undergo a transient increase in cytokine production, but instead acquire an epigenetic framework that is more readily reactivated upon subsequent challenge [213]. Consistent with this,​ in macrophages derived from IL-10^−/−^ mice and in a microbiota-dependent colitis model, the bromodomain and extraterminal (BET) inhibitor (+)-JQ1 suppresses inflammatory activation and constrains the associated chromatin opening [214]. Although there is still no direct evidence demonstrating that each individual metabolic–epigenetic node has been validated stepwise in mucosal macrophages from patients with IBD, studies in a β-glucan induced trained immunity model of human monocytes have shown that enhanced glycolysis driven by the Dectin-1–Akt–mTOR–HIF-1α axis is accompanied by remodeling of H3K4me3 and H3K27Ac, whereas glutaminolysis, fumarate accumulation, and the mevalonate–IGF1R–mTOR axis also contribute to the establishment and maintenance of a hyperresponsive state [215–217].

At the T cell level, the relapse tendency of IBD is first reflected by the incomplete disappearance of the proinflammatory memory pool after clinical remission. Combined cytometry by time of flight (CyTOF) and single-cell RNA sequencing (scRNA-seq) analyses showed that, compared with healthy controls, CCR6^+^TNF^+^CD161^+^ effector memory T cells remained enriched in the mucosa of patients with UC during both active disease and remission, whereas active disease was additionally associated with increased TNF^+^IL^−^17A^+^ effector memory Treg [218]. Moreover, this persistence is not merely quantitative residue, but can be further amplified by local tissue residency. In the intestinal mucosa of patients with active Crohn’s disease, CD4^+^ tissue resident memory T cells are markedly expanded and represent a major source of mucosal TNF-α. At the same time, resident intestinal CD8^+^ T cell subsets defined by KLRG1 and CD103 display distinct inflammatory potential, among which the CD103^+^ subset can induce tissue wide alarm-like responses and enhance innate immune cell recruitment [219, 220]. These findings suggest that reactivation of resident T cells may contribute to recurrent inflammation, but do not establish that it predominates over newly recruited cells in human relapse [219, 220]. In line with this, in chronic enteritis models, Hobit and Blimp-1 jointly drive the program of CD4^+^ tissue resident memory T cells and sustain chronic intestinal inflammation, whereas a long lived memory-like subset derived from lamina propria effector memory CD4^+^ T cells can survive long term under IL-7 support, suggesting that prior local inflammatory experience can be written into relatively durable programs of T cell residency and survival [221, 222]. In mice with Treg-specific deletion of *Tfam*, loss of mitochondrial transcription factor A in Treg increases methylation of the Treg specific demethylated region at the *Foxp3* locus, impairs Treg homing and stability, and results in failure of inflammatory control in colitis models, indicating that mitochondrial metabolic status can influence Treg lineage maintenance through epigenetic mechanisms [223].

Human IBD cohort studies further showed that the transcriptional behavior of Treg and CD4^+^ memory T cells differs more strongly between the ileum and colon than between inflammatory states per se, and that response to vedolizumab is associated with upregulation of genes related to oxidative phosphorylation in Treg, suggesting that the local anatomical environment and metabolic adaptation jointly shape T cell states [224]. Consistently, Medina and colleagues showed that in intestinal mucosal CD4 T cells from patients with IBD, the open chromatin landscape of inflamed biopsies is predominantly linked to inflammatory gene networks involved in lymphocyte activation, differentiation, and proliferation, and is associated with enrichment of pathogenic Th17 related programs, whereas noninflamed mucosa and peripheral blood CD4 T cells do not exhibit the same inflammation associated chromatin features. These findings link local T-cell effector programs to chromatin accessibility; whether these features persist after inflammation resolves requires longitudinal validation [225]. At the same time, Gonzalez and colleagues demonstrated that BMI1 is required to maintain the epigenomic landscape of Treg, and that BMI1 deficiency drives Treg toward a Th1-like and Th17-like proinflammatory phenotype and promotes colitis [226]. Together with the single cell and spatial findings of Garrido-Trigo and colleagues in human inflamed intestinal tissue, in which the most prominent interpatient differences were concentrated in the myeloid compartment, these data support a model in which IBD relapse is more likely to arise from the superimposition of persistent metabolic–epigenetic priming in myeloid cells and local reactivation of resident memory T cells within the tissue framework, rather than from a completely independent de novo inflammatory event [227].

## Therapeutic reprogramming of metabolism and epigenetics

Chronic relapse in IBD is tightly coupled to metabolic adaptation in immune cells. Recent studies link drug efficacy to reprogramming of glucose, lipid, and amino-acid metabolism as well as to epigenetic plasticity. Clinical and translational work indicates that redirecting immune-cell fate through defined receptors and metabolic circuits, or remodeling inflammatory transcriptional programs via epigenetic enzymes, may complement current anti-inflammatory and biologic therapies. Several compounds have already been evaluated in randomized trials or pilot clinical testing, building an actionable chain of evidence [228, 229]. Below, agents are organized by metabolic axis. For each, we summarize the dose and study design, applicable populations or models, clarified molecular pathways, and efficacy findings or limitations. A consolidated overview appears in Table 2.

**Table 2:** Clinical and preclinical interventions targeting metabolic and epigenetic pathways in IBD

Drug	Dose	Species or study type	Mechanisms and main findings	Reference
Metformin	Oral 1000 mg per day in divided doses for 24 weeks	Human randomized double-blind trial in mild to moderate ulcerative colitis	Lowers clinical activity index and improves endoscopic mucosa, indicating metabolic anti-inflammatory effect	[241]
Metformin	100 to 200 mg per kg in DSS mouse model	Mouse	Activates AMPK, suppresses STAT3 and IL-17, increases Treg and decreases Th17, achieving immune metabolic reprogramming	[242]
Metformin	Not reported in this mechanistic paper	In vitro and human samples	Inhibits FASN, reduces Akt palmitoylation and membrane localization, downregulates NF-κB and MAPK driven pro inflammatory transcription	[243]
Dichloroacetate	5 to 100 mg per kg orally or intraperitoneally	Mouse and in vitro T cells	Promotes Treg differentiation and suppresses Th17 differentiation through a reported PDHK-independent mechanism.	[244]
Dichloroacetate	Not reported	Mouse and in vitro human cells	PDHK1-dependent metabolic regulation of CD4 + T-cell differentiation and function in experimental inflammatory models.	[245]
Rosiglitazone	4 mg twice daily for 12 weeks	Human multicenter randomized double-blind trial in mild to moderate ulcerative colitis	PPARγ activation suppresses NF-κB related inflammatory transcription and improves clinical and endoscopic outcomes	[229]
Rosiglitazone	4 mg twice daily for 12 weeks	Human open label study	PPARγ activation improves clinical or endoscopic outcomes, individualized safety monitoring advised	[246]
Rosiglitazone mechanism	Not reported	In vitro macrophage and related models	PPARγ SUMOylation recruits NCoR-HDAC3 corepressor complex to keep promoters deacetylated, leading to transrepression of NF-κB	[247]
Sirolimus	Individualized dosing with target trough 5 to 10 ng per mL	Retrospective study in children with refractory IBD	mTORC1 inhibition decreases effector T cell metabolism and proliferation and enhances autophagy and barrier related signaling	[248]
Sirolimus mechanism	Not reported	Mouse and in vitro	Upregulates PBLD and stabilizes AMOT to promote epithelial barrier repair with decreased inflammation scores	[249]
Butyrate enema	80 mmol per L, 60 mL nightly for 6 weeks	Human randomized controlled trial in left sided ulcerative colitis	Epigenetic HDAC inhibition and energy substrate function, trial not superior to placebo	[255]
Short chain fatty acid enema	Not specified in that study	Human small randomized trial	Restoration of SCFA signaling, symptom and endoscopic improvements reported with protocol heterogeneity	[256]
Butyrate mechanism	Not reported	Mouse	HDAC inhibition increases *Foxp3* locus acetylation and induces colonic Tregs with enhanced suppressive function	[14]
SCFA mechanism	Not reported	Mouse	GPR43 activation mediates inflammation resolution and promotes immune tolerance	[257]
Resveratrol	500 mg once daily for 6 weeks	Human randomized double-blind trial in mild to moderate ulcerative colitis	Activates SIRT1 and inhibits NF-κB, reduces TNF-α and hsCRP, lowers PBMC NF-κB activity, improves quality of life	[258]
Curcumin maintenance	2 g per day in divided doses for 6 months	Human multicenter randomized double-blind trial in remission maintenance	Inhibits NF-κB and modulates antioxidant and epigenetic pathways, reduces relapse rate and improves endoscopic scores	[259]
Vidofludimus	Oral 35 mg once daily for 12 weeks	Human open-label uncontrolled study in steroid-dependent IBD	Among 26 patients evaluable for the primary efficacy analysis, steroid-free clinical remission was achieved in 8/14 patients with CD and 6/12 patients with UC; vidofludimus is a DHODH inhibitor and also inhibits expression of IL-17A, IL-17F, and IFN-γ.	[250]
Vidofludimus calcium (IMU-838)	10, 30, or 45 mg once daily for 10 weeks	Human randomized placebo-controlled phase II trial in moderately to severely active UC	As a follow-on DHODH inhibitor, vidofludimus calcium did not improve induction-phase clinical remission versus placebo in the overall study population; 16/113 patients (14%) in the pooled 30/45 mg groups achieved week-10 clinical remission versus 8/56 (14%) with placebo.	[252]
NX-13	250 mg immediate-release, 500 mg immediate-release, or 500 mg delayed-release once daily for 4 weeks	Human randomized double-blind placebo-controlled phase Ib study in active UC	NX-13 is an NLRX1 agonist; in the efficacy population, improvements in rectal bleeding and stool frequency relative to placebo were seen by week 2, and an endoscopic response was observed by week 4.	[253]
NX-13	Oral administration; dosing varied by model	Mouse DSS colitis, Mdr1a-deficient colitis, and CD45RBhi adoptive transfer model; with in vitro naïve CD4^+^ T-cell experiments	NLRX1 activation reduced disease severity, colonic leukocyte infiltration, and inflammatory cytokine levels; in naïve CD4^+^ T cells, NX-13 reduced Th1/Th17 differentiation, reactive oxygen species, and NF-κB activation, while enhancing oxidative phosphorylation.	[254]
CKD-506	Oral 10 or 30 mg/kg/day in the DSS model; oral administration in chronic models	Mouse acute and chronic colitis models; with intestinal epithelial cells and macrophages in vitro	CKD-506 is a selective HDAC6 inhibitor that suppressed NF-κB signaling and pro-inflammatory cytokine production in intestinal epithelial cells and macrophages, and ameliorated DSS-induced acute colitis as well as chronic colitis in *Il10*^−/−^ and adoptive transfer models.	[143]
CKD-506	Not reported in this ex vivo study	Human PBMCs and CD4^+^ T cells from patients with IBD; with cell line and colon organoid assays	Anti-inflammatory effects were observed in patient-derived PBMCs and CD4 + T cells. Separate cell-line and colon-organoid assays assessed improvements in barrier-related readouts.	[261]

### Immune and epigenetic biomarkers for IBD stratification

In a CyTOF and scRNA-seq analysis of active and inactive UC, mucosal CCR6^+^TNF^+^CD161^+^ effector memory T cells were enriched in both groups relative to healthy controls, whereas CD38^+^TNF^+^ effector memory Treg cells and HLA-DR^+^CD14^+^IL-21^+^ macrophages or monocytes were more characteristic of active mucosa [218]. By contrast, HLA-DR^+^IL-21^+^ macrophages or monocytes were more enriched in peripheral blood during inactive disease, indicating that UC stratification can capture not only persistently shifted T-cell memory phenotypes but also myeloid activation states that fluctuate with disease activity [218]. In a combined imaging mass cytometry and scRNA-seq analysis of human UC colonic tissue, the macrophage disappearance reaction occurred predominantly in tissue-resident macrophages, and lesional areas were characterized by increased ROS, insufficient SOD2 expression in resident macrophages, an increase in inflammatory macrophages, and a spatial shift in the dominant source of TNF from resident to inflammatory macrophages, indicating that spatially resolved changes in myeloid states can likewise serve as indicators of local histologic inflammatory stratification [218]. With respect to treatment-response stratification, low TREM-1 expression in peripheral blood CD14^+^ monocytes before treatment in patients with CD was associated with subsequent anti-TNF response, whereas TREM-1^high^ monocytes were less able to differentiate into CD206^+^ macrophages with regulatory features under anti-TNF exposure and showed reduced Fcγ receptor activity and autophagy, suggesting that this marker is linked not only to therapeutic efficacy but also to differences in monocyte-macrophage differentiation plasticity [230]. Further, a single-cell atlas of ileal CD lesions defined the GIMATS module, composed of IgG plasma cells, inflammatory mononuclear phagocytes, activated T cells, and stromal cells. Across four independent cohorts, this module was associated with failure to achieve sustained steroid-free remission after anti-TNF therapy, suggesting that more robust stratification markers may need to reflect cross-cellular networks and tissue-level immune states rather than remaining confined to a single cell type or single mediator [231].

At the epigenetic level, 850K methylation profiling of peripheral blood in the pediatric CD RISK cohort showed that most disease-associated CpG methylation changes present at diagnosis were attenuated after anti-inflammatory treatment or shifted toward control levels [232]. By contrast, in sorted peripheral blood CD14^+^ monocytes from patients with active or inactive CD and from non-CD controls, differential methylation was localized mainly to promoter regions of inflammation-related genes, suggesting that focusing methylation analysis on the monocyte-macrophage lineage may provide greater resolution for disease state and activity [233]. In a longitudinal pediatric UC rectal mucosal cohort, cell-type-specific DNA methylation was associated with disease state, severity, and clinical outcome; post-treatment methylation changes were more pronounced in immune cells, whereas epithelial and fibroblast alterations were relatively persistent, and patients who underwent colectomy retained cell-composition and methylation profiles at follow-up that were closer to those at active baseline [234]. At the T-cell level, transcriptomic studies of peripheral blood CD8^+^ T cells in adult CD and UC first suggested that T-cell state was associated with subsequent disease course, and this information was subsequently translated into a 17-gene whole-blood qPCR classifier that stratified newly diagnosed patients with IBD into subgroups at high or low risk of treatment escalation [235, 236]. However, in an independent pediatric CD cohort, although transcriptional and DNA methylation patterns in blood-derived CD8^+^ T cells were associated with age and IBD status, they failed to reproduce the prognostic stratification observed in adults, indicating that classifications based on T-cell epigenetic or transcriptional states still require external validation across age groups and disease spectra [237]. For drug-response stratification, longitudinal multi-omics studies in IBD have shown that early changes in peripheral blood gene expression and DNA methylation can predict anti-TNF efficacy, and whole-blood methylation analysis of 385 patients with CD in the PANTS cohort further refined this framework by identifying 323 differentially methylated sites associated with week-14 drug concentrations, some of which also overlapped with primary non-response and mapped to inflammatory signaling nodes including SOCS3 and STAT3 [238, 239]. In moderate-to-severe UC, peripheral blood DNA methylation signatures likewise predicted response to tofacitinib [240].

### Metabolism-targeted strategies

**Metformin.** In a randomized, double-blind trial in mild to moderate active UC, oral metformin at 1000 mg per day in divided doses as add-on therapy for 24 weeks significantly reduced disease indices and improved endoscopic mucosal appearance, suggesting anti-inflammatory potential in non-diabetic patients [241]. In DSS colitis, effective doses most commonly range from 100 to 200 mg per kg, mitigating weight loss and histologic inflammation. Mechanistically, metformin activates AMP-activated protein kinase (AMPK), inhibits STAT3 signaling, and downregulates IL-17, with a reciprocal increase in Treg and decrease in Th17 frequencies [242]. In macrophages, metformin inhibits fatty-acid synthase (FASN) to reduce Akt palmitoylation, weakening Akt membrane localization and activation and thereby attenuating NF-κB and MAPK-driven proinflammatory transcription. Mucosal samples from patients show concordant suppression of inflammatory pathways [243]. Clinically, evidence currently supports add-on use in mild to moderate UC with attention to gastrointestinal intolerance.

**Dichloroacetate.** Dichloroacetate has been studied experimentally as a modulator of T-cell metabolism and differentiation. One study reported increased Treg differentiation and reduced Th17 differentiation through a mechanism independent of pyruvate dehydrogenase kinase (PDHK) inhibition [244]. The cited studies also implicate PDHK1-dependent metabolic regulation in CD4 + T-cell subsets and experimental inflammation [244, 245]. The reported effects therefore involve distinct experimental mechanisms. These studies do not establish clinical efficacy or a dosing regimen for patients with IBD.

**Rosiglitazone.** As a PPARγ agonist, rosiglitazone suppresses inflammation through combined metabolic and transcriptional mechanisms. In a multicenter, randomized, double-blind trial, rosiglitazone at 4 mg twice daily for 12 weeks increased clinical response and remission rates compared with placebo in patients with mild-to-moderately active UC; endoscopic remission was uncommon and did not differ significantly between groups [229]. An earlier 12-week open-label study using the same dose reported clinical and endoscopic remission in some patients. Two patients were hospitalized for worsening disease activity, and one withdrew because of nephrotic syndrome [246]. Mechanistically, ligand-dependent SUMOylation of PPARγ tethers it to the NCoR–HDAC3 corepressor complex at inflammatory promoters, maintaining deacetylation and transcriptional repression to counter NF-κB-driven programs in colonic mucosa [247].

**Sirolimus.** By inhibiting mTORC1, sirolimus reduces glycolysis and protein-synthesis demands in effector T cells and is used in practice as rescue therapy for refractory pediatric IBD, with individualized dosing to achieve troughs of 5 to 10 ng per mL and adjustments by body surface area or weight plus therapeutic drug monitoring [248]. Beyond immunosuppression, mTOR inhibition promotes autophagy and tight-junction programs. Recent data indicate that sirolimus upregulates phenazine biosynthesis-like protein domain-containing protein (PBLD) and stabilizes angiomotin (AMOT) to enhance epithelial repair, reduce inflammation scores, and increase barrier-protein expression, suggesting benefits for mucosal healing that extend beyond immune dampening [249]. Systematic adult data remain limited, but pediatric cohorts after anti-TNF failure show clinical improvement and steroid-sparing potential; hyperlipidemia and aphthous ulcers are the main adverse events, supporting therapeutic drug and metabolic monitoring to maximize safety [248, 249].

**Vidofludimus**. Early evidence suggested that the dihydroorotate dehydrogenase (DHODH) inhibitor vidofludimus had some steroid-free remission potential in steroid-dependent IBD. In an open-label study of patients with steroid-dependent IBD treated with oral vidofludimus 35 mg once daily for 12 weeks, steroid-free clinical remission was achieved in 8 of 14 patients with Crohn’s disease and 6 of 12 patients with ulcerative colitis among the 26 patients evaluable for the primary efficacy analysis [250]. Its follow-on compound, vidofludimus calcium, also known as IMU-838, was subsequently evaluated in healthy male subjects in single ascending oral dose studies of 10–40 mg and multiple ascending oral dose studies of 30–50 mg. A subsequent phase II study in patients with moderately to severely active ulcerative colitis used 10, 30, or 45 mg once daily for 10 weeks, and the pooled 30 or 45 mg groups showed a week 10 clinical remission rate of 14%, which was identical to that in the placebo group [251, 252]. Mechanistically, vidofludimus inhibits DHODH and downregulates the expression of pro-inflammatory cytokines such as IL-17A, IL-17F, and IFN-γ, suggesting that it may exert immunomodulatory effects by interfering with de novo pyrimidine synthesis in activated lymphocytes and with Th17- and Th1-associated inflammatory output [250].

**NX-13**. In a phase Ib study in active ulcerative colitis, NX-13 was administered once daily for 4 weeks as 250 mg immediate-release, 500 mg immediate-release, or 500 mg delayed-release formulations. Compared with placebo, improvements in rectal bleeding and stool frequency subscores were observed as early as week 2, and an endoscopic response was observed by week 4 [253]. In three IBD models, namely DSS colitis, Mdr1a-deficient colitis, and the CD45RB^hi^ adoptive transfer model, oral NX-13 reduced disease severity, colonic leukocyte infiltration, and inflammatory cytokine levels [254]. Mechanistically, upon activation of NLR family member X1 (NLRX1), NX-13 inhibited the differentiation of naive CD4^+^ T cells into Th1 and Th17 subsets in vitro, accompanied by reduced reactive oxygen species production and NF-κB activation together with enhanced oxidative phosphorylation, suggesting that its effects are associated with immunometabolic reprogramming of CD4^+^ T cells [254].

### Epigenetics-informed small molecules and microbial metabolites

**Short-chain fatty acids (SCFAs).** As microbiota-derived epigenetic modulators, short-chain fatty acids inhibit HDAC and serve as energy substrates in epithelium and immune cells. In left-sided UC, nightly 60 mL enemas of sodium butyrate at 80 mmol per L for six weeks were not superior to placebo, indicating limited efficacy of single-agent topical delivery in active distal disease [255]. In a randomized trial of mixed SCFA enemas, overall improvement did not differ significantly from placebo, although patients with shorter episodes of active disease appeared to benefit [256]. Mechanistically, butyrate induces colonic Treg and strengthens suppressive function via HDAC inhibition and increased acetylation at the *Foxp3* locus, and through G-protein–coupled receptor 43 (GPR43) mediates granulocyte–macrophage braking, establishing a metabolism–epigenetics–tolerance axis [14, 257].

**Resveratrol.** This pleiotropic anti-inflammatory and epigenetic modulator activates SIRT1 and suppresses NF-κB transcriptional activity. In a randomized double-blind trial in patients with mild-to-moderately active UC, 500 mg once daily for six weeks reduced TNF-α and high-sensitivity C-reactive protein, lowered NF-κB activity in peripheral mononuclear cells, increased nine-item IBD Questionnaire (IBDQ-9) scores, and decreased disease activity, indicating clinical benefit through systemic epigenetic anti-inflammation with favorable short-term safety [258]. Given its profile, resveratrol is best positioned as an adjunct rather than a replacement for standard care. Longer studies should test impacts on mucosal healing and endoscopic endpoints [258].

**Curcumin.** With metabolic and epigenetic actions that include NF-κB inhibition, curcumin has supportive evidence in both maintenance and induction. In maintenance, 2 g per day in divided doses combined with mesalamine for six months significantly reduced relapse rates, supporting feasibility as an add-on in remission [259]. For induction, adding curcumin to mesalamine for 4 weeks improved clinical and endoscopic remission in mild-to-moderate active UC, with good tolerability and no serious drug-related adverse events reported [260].

**CKD-506**. CKD-506 currently lacks published efficacy data from oral treatment studies in patients with IBD, and the available evidence for IBD-related benefit is derived mainly from orally administered mouse models of acute and chronic colitis, in which it attenuated colitis severity [143]. In PBMCs and CD4 + T cells derived from patients with IBD, CKD-506 exerted anti-inflammatory effects. Separate cell-line and colon-organoid experiments evaluated its effects on gut barrier-related readouts [261]. Mechanistically, as an HDAC6 inhibitor, CKD-506 suppresses NF-κB signaling in intestinal epithelial cells and macrophages, and reduces inflammatory responses in human PBMCs and CD4-positive T cells, suggesting that its effects are associated with HDAC6-dependent immunoregulation involving both macrophages and T cells [143, 261].

## Conclusion and future perspectives

This review synthesizes how metabolic reprogramming and epigenetic regulation shape the immune microenvironment in IBD. We argue that the central therapeutic challenge in IBD is to reshape metabolic and epigenetic programs that sustain inflammatory responsiveness while preserving those required for immune regulation and mucosal repair. Diet-induced alterations in bile acid metabolism can contribute to colitis initiation in genetically susceptible mice. After inflammation develops, local hypoxia and associated glycolytic reprogramming can sustain immune activation and amplify intestinal inflammation. Treg depend on oxidative metabolism, yet their trafficking and function require appropriate glycolytic power. These mechanisms share a dependence on the responding cell and its functional state. Glycolysis supports both inflammatory activation and Treg migration, while histone acetylation can promote inflammatory gene expression or regulatory programs at the *Foxp3* locus. The therapeutic implication is to identify dependencies of pathogenic cell states and determine whether they can be targeted while maintaining regulatory function. The persistence of altered mucosal T-cell populations during remission, together with experimental evidence of myeloid reprogramming, raises a further question: whether metabolic and epigenetic changes preserve inflammatory responsiveness after disease activity subsides. We propose that the reversibility of these residual immune states should guide the development of strategies for sustained remission.

Future work should determine whether metabolic and epigenetic features retained in mucosal macrophages and T cells during remission predict subsequent relapse. Longitudinal sampling combined with single-cell and functional analyses could identify which persistent features contribute to renewed inflammation and whether modifying them preserves regulatory and repair functions. For the palmitoylation mechanisms discussed in this review, a priority is to establish whether NLRP3 and STAT3 palmitoylation is altered in human IBD mucosal immune cells. Comparisons of inflamed and noninflamed mucosa, with non-IBD controls, should measure palmitoylation of the target proteins in macrophages and CD4^+^ T cells and relate these changes to inflammasome activation and STAT3-dependent inflammatory responses, respectively. This would establish whether the mechanisms identified in experimental models are present at sites of human intestinal disease. Functional studies in patient-derived mucosal cells should then test whether perturbing the implicated palmitoyltransferases or APT2 changes target-protein palmitoylation and the corresponding inflammatory response. For NLRP3, the activating and inhibitory effects reported for different palmitoylation sites make enzyme- and site-specific validation necessary. These experiments would clarify which components of the palmitoylation network are relevant therapeutic targets in human IBD and whether their modulation can reduce inflammation while preserving regulatory function.

## Data Availability

No datasets were generated or analysed during the current study.
